# The clove (*Syzygium aromaticum*) genome provides insights into the eugenol biosynthesis pathway

**DOI:** 10.1038/s42003-022-03618-z

**Published:** 2022-07-09

**Authors:** Sonia Ouadi, Nicolas Sierro, Simon Goepfert, Lucien Bovet, Gaetan Glauser, Armelle Vallat, Manuel C. Peitsch, Felix Kessler, Nikolai V. Ivanov

**Affiliations:** 1 Faculty of Sciences, Laboratory of Plant Physiology, University of Neuchâtel, 2000 Neuchâtel, Switzerland; 2 PMI R&D, Philip Morris Products S. A, Quai Jeanrenaud 5, CH-2000 Neuchâtel, Switzerland; 3 Faculty of Sciences, Neuchâtel Platform of Analytical Chemistry, University of Neuchâtel, 2000 Neuchâtel, Switzerland

**Keywords:** Comparative genomics, Secondary metabolism

## Abstract

The clove (*Syzygium aromaticum*) is an important tropical spice crop in global trade. Evolving environmental pressures necessitate modern characterization and selection techniques that are currently inaccessible to clove growers owing to the scarcity of genomic and genetic information. Here, we present a 370-Mb high-quality chromosome-scale genome assembly for clove. Comparative genomic analysis between *S. aromaticum* and *Eucalyptus grandis*—both species of the Myrtaceae family—reveals good genome structure conservation and intrachromosomal rearrangements on seven of the eleven chromosomes. We report genes that belong to families involved in the biosynthesis of eugenol, the major bioactive component of clove products. On the basis of our transcriptomic and metabolomic findings, we propose a hypothetical scenario in which eugenol acetate plays a key role in high eugenol accumulation in clove leaves and buds. The clove genome is a new contribution to omics resources for the Myrtaceae family and an important tool for clove research.

## Abstract (web summary)

A newly assembled clove genome is compared with *E. grandis* to investigate genome evolution between the two genera of the Myrtaceae family, and putative genes involved in the biosynthesis of eugenol are identified through transcriptomics and metabolomics.

## Introduction

Clove, *Syzygium aromaticum* (L.) Merr. & L.M. Perry, is a perennial tropical tree that belongs to the Myrtaceae family (which also includes myrtle, eucalyptus, and guava). Native to the Maluku Islands in eastern Indonesia, also known as the “spice islands”, clove is one of the most anciently used and traded spices^1^. Beginning in the 18th century, clove trees were introduced to different parts of the world, where they became economically important^2,3^. Cultivated for its dried, unopened flower buds (cloves), oleoresin and essential oil (EO) rich in eugenol, the clove is one of the most important tropical spice crops in global trade. Its products are commercially used in a wide range of applications within the pharmaceutical, cosmetic, food, tobacco, and agricultural industries^4–7^.

The Myrtaceae family is the eighth largest family of flowering plants. It includes more than 5650 species, distributed across ~130 to 140 genera. The majority of species are diploid (2n = 22), with small to intermediate genome sizes (234–1788 Mb)^8,9^. The chromosome-level genome assembly of *Eucalyptus grandis* (640 Mb) is a reference genome for all species that belong to the Myrtaceae family and is a valuable tool for comparative genomics^10–14^. Clove is the most commercialized species of the species-rich (1200–1500 species) and highly diversified genus *Syzygium*, which comprises other interesting woody species cultivated for their edible fruits, timber, and medicinal properties (e.g., *Syzygium cumini* and *Syzygium jambos*)^15,16^. Previous karyotype studies indicate that clove is a diploid with 2n = 22 chromosomes^17^. However, its genome size has not been recorded in the C-value database and no reference genome or genetic map is currently available for clove^9^. A reference genome for clove would be fundamental to start investigating the genetic basis of economically important traits related to yield (such as organ size, disease resistance), cost of harvesting (low branching and falling buds), and biosynthesis of secondary compounds that are important for the quality of clove products.

The major compounds found in commercial-type clove EO are eugenol (72–96.6%), eugenol acetate (0–21.3%), β-caryophyllene (1.7–19.5%), and α-humulene (0.2–2.2%)^18^. Eugenol (4-allyl-2-methoxyphenol) is the main bioactive compound of clove and is a versatile compound with analgesic, antioxidant, anti-inflammatory, antibacterial, antifungal, and antiviral activities^19–21^. Eugenol belongs to the phenylpropene group, a large group of phenylpropanoid volatiles that contributes to the aroma and flavor of spices, herbs, and fruits and plays a role in plant communication (pollinator attraction) and defense (against insects, herbivores, and pathogens)^22,23^. The biosynthesis of eugenol, from the phenylalanine produced by the plant shikimate pathway, involves gene families of the general phenylpropanoid pathway and the branch of the phenylpropanoid pathway specific to lignin biosynthesis for producing monolignol coumaryl alcohol and coniferyl alcohol (CCR, cinnamoyl CoA reductase, and CAD, cinnamyl alcohol dehydrogenase). The final steps are catalyzed by biosynthetic genes belonging to families containing some members involved in the production of volatile phenylpropenes (BAHD (benzyl alcohol-acetyl-, anthocyanin-*O*-hydroxy-cinnamoyl-, anthranilate-N-hydroxycinnamoyl/benzoyl-, deacetyl-vindoline) acyltransferase superfamily and PIP (pinoresinol–lariciresinol reductase, isoflavone reductase, phenylcoumaran benzylic ether reductase) family)^23–25^. The biosynthetic pathway leading to the production of phenylpropene volatiles has been studied in several herbs and fruits, and many biosynthetic genes have been isolated and characterized^25^. Although clove products are considered the richest source of eugenol, the biological and molecular mechanisms involved in the biosynthesis, high accumulation, and regulation of eugenol in clove organs have not been extensively investigated.

Here, we report a high-quality reference genome for clove assembled into eleven chromosomes. In this study, we compared the newly assembled clove genome with the genome of *E. grandis* to investigate genome evolution between the two genera of the Myrtaceae family. In addition, we analyzed the genome to identify key gene families involved in the biosynthesis of eugenol and performed a combined transcriptomic and metabolomic analysis to gain insights into eugenol biosynthesis and its accumulation in clove leaves and buds.

## Results

### Genome de novo assembly and annotation

By using Illumina paired-end (PE) raw reads and the K-mer analysis software GenomeScope 2.0, the clove genome size was estimated to be 343 Mb (k = 21 bp)^26^ (Supplementary Fig. 1). The use of third-generation long-read sequencing technologies from Oxford Nanopore Technologies (ONT) combined with Hi-C technology enabled de novo assembly of a high-quality reference genome for clove (Supplementary Data 1). The final assembly resulted in a 370 Mb genome consisting of 24 scaffolds, with 99.3% of the assembly being anchored on the 11 chromosomes (Table 1) (Supplementary Table 1). The completeness of the *S. aromaticum* genome assembly was assessed by using BUSCO, with the eudicots_odb10 dataset^27^. Of the tested BUSCO genes, 98.2% were found in a complete form, a percentage similar to that observed in the eucalyptus genome (97.9%). The low percentage of missing BUSCO genes (1.00%) suggests that the clove genome assembly is nearly complete (Supplementary Fig. 2).

**Table 1:** The statistics for genome assembly of *S. aromaticum*.

	Chromosome-level assembly
Assembly	
Number of scaffolds	24
Minimum length of scaffolds (bp)	11,734
Maximum length of scaffolds (bp)	43,763,418
Average length of scaffolds (bp)	15,427,406.3
N	36,000 (0.01%)
N50 (bp)	35,418,074
Quality value of the assembly (QV)	46.738
Number of chromosome-scale scaffolds	11
Total length of chromosome-scale scaffolds	367,665,948 (99.3%)
Total length of assembly (bp)	370,257,752
Annotation	
Number of predicted genes	27,528
Number of predicted transcripts	50,053
Average transcript length (bp)	2,215.27
Average CDS length (bp)	1,277.41
Average exon per transcript	6.28
Annotated with UniRef Malvids	22,139 (80%)
Repeat sequences (bp)	160,689,965 (43.4%)
LTR retrotransposons (bp)	80,731,053 (21.80%)
LTR/Copia (bp)	29,874,465 (8.1%)
LTR/Gypsy (bp)	48,891,039 (13.2%)
Others (bp)	79,958,912 (21.6%)

By using RNAseq data from clove and transcripts of eucalyptus, a total of 27,528 protein-coding genes were predicted, representing 31% (115 Mb) of the genome assembly (Supplementary Data 1). The BUSCO results indicated high coverage of the gene space by the gene model and protein predictions: 95% of BUSCOs at the transcript level and 93.7% of BUSCOs at the protein level were found in complete form by using the eudicots_odb10 ortholog dataset (Supplementary Fig. 2). The clove genome comprises 43.4% (161 Mb) repetitive DNA, a fraction similar to that reported in the Myrtaceae relative guava (*Psidium guajava*) 443.8 Mb genome assembly (43.55%)^14^. Among the identified transposable elements, the long terminal repeats retrotransposons (LTR-RTs) represented 21.80% of the clove assembly. The LTR-RTs superfamilies Copia and Gypsy LTR-RTs were the most abundant repeat elements, representing 8.1% (30 Mb) and 13.2% (49 Mb) of the genome assembly, respectively (Table 1).

### Clove genome evolution

**Fig. 1:**
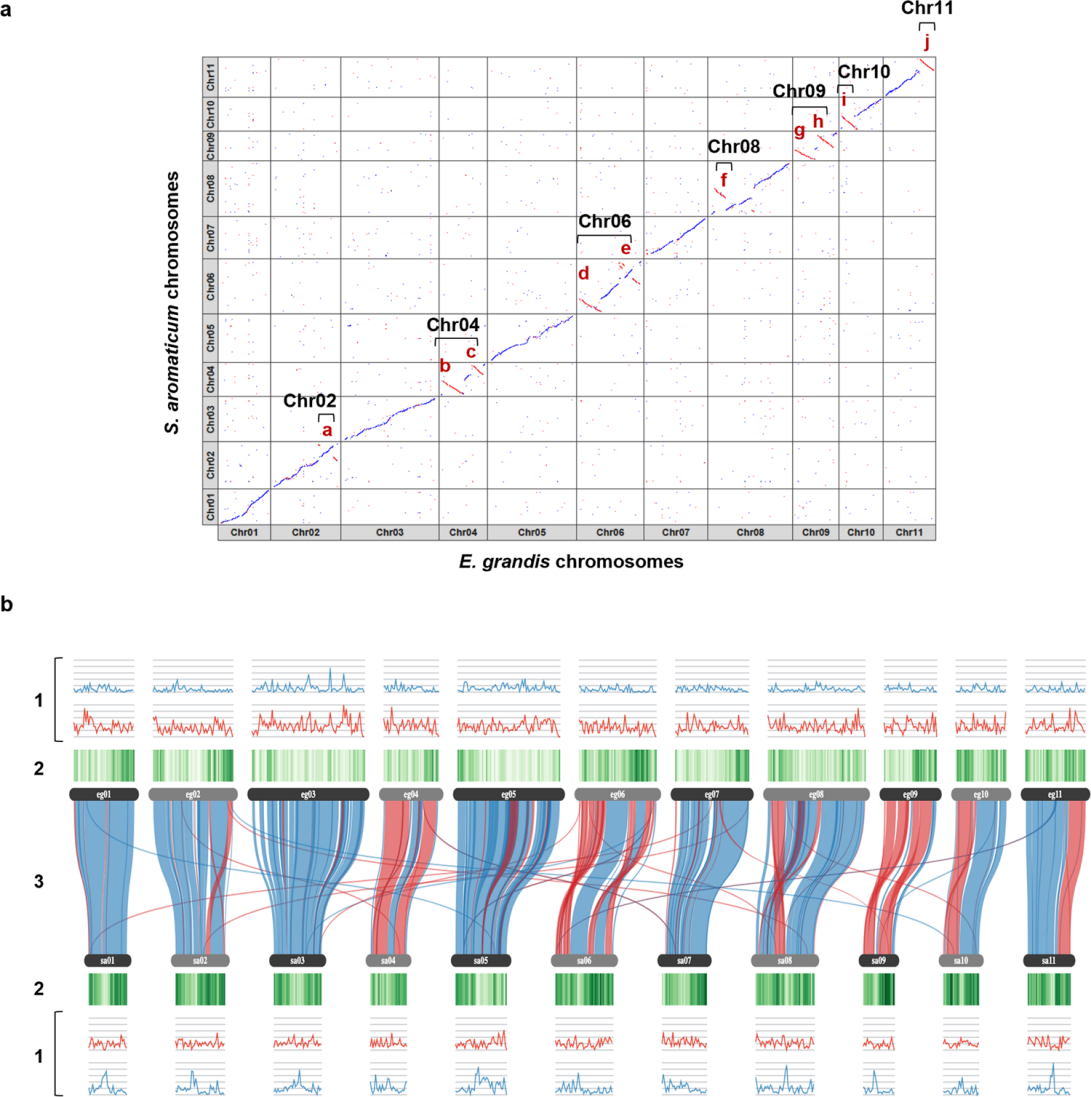
Comparative analysis of the *Syzygium aromaticum* genome with *Eucalyptus grandis* genome. **a** DNA alignment of the *S. aromaticum* and *E. grandis* 11 chromosomes. The alignment of the DNA sequences of *E. grandis* and *S. aromaticum* chromosomes (Chr) is shown in blue. The main intrachromosomal rearrangements (a to j) are indicated in red. **b** SynVisio representation of *E*. *grandis* (eg) and *S*. *aromaticum* (sa) chromosomes showing the (1) distributions of Copia (red) and Gypsy (blue) LTR retrotransposons; (2) density of protein-coding genes; and (3) alignment of protein syntenic blocks between the two Myrtaceae species.

According to our comparative analysis of the clove genome with the eucalyptus genome, no major interchromosomal rearrangements were observed between the two Myrtaceae species (Fig. 1). The structures of chromosomes 1, 3, 5, and 7 are highly conserved between *E. grandis* and *S. aromaticum*, and ten intrachromosomal rearrangements (a to j) were found on the other seven chromosomes 2, 4, 6, 8, 9, 10, and 11. Large terminal inversions are present on chromosomes 4, 6, 9, 10, and 11, and more complex terminal rearrangements occur on chromosomes 2, 6, and 8 (Supplementary Table 2).

To determine if the rearrangements detected in the *S. aromaticum* genome assembly might be due to our assembly pipeline, we verified the Hi-C signal among the *S. aromaticum* chromosomes on the Hi-C contact maps. The strong Hi-C intrachromosomal signal among the *S. aromaticum* chromosomes and the very low interchromosomal Hi-C signal implied that there is a high number of Hi-C data supporting the proposed scaffolding of contigs into the 11 *S. aromaticum* chromosomes (Supplementary Fig. 3). To further investigate the reliability of the proposed *S. aromaticum* chromosomes scaffolding and the intrachromosomal rearrangements found between the chromosomes of *S. aromaticum* and *E. grandis*, we compared our results to those from a previous comparative study between the high-density linkage map of the *Corymbia citriodora* subsp. *variegata* (370 Mb) and *E. grandis* genome (640 Mb)^28^. Interestingly, the structures of chromosomes 1, 3, 5, and 7 are highly conserved between *E. grandis* and *S. aromaticum*, as are those between the two eucalypt species. The presence of intrachromosomal rearrangements was found between *S. aromaticum* and *E. grandis* and between *C. citriodora subsp. variegata* and *E. grandis* was detected on the other seven chromosomes, chromosomes 2, 4, 6, 8, 9, 10, and 11. The same type of rearrangement—large terminal inversions—was found on chromosomes 4 (c), 9 (h), 10 (i), and 11 (j) of *C. citriodora* subsp. *variegata* and *S. aromaticum* when compared to *E. grandis*. Two additional large terminal inversions were detected on *S. aromaticum* chromosomes 4 (b) and 9 (g) but not in the *C. citriodora* subsp. *variegata* linkage map (Fig. 1).

**Fig. 2:**
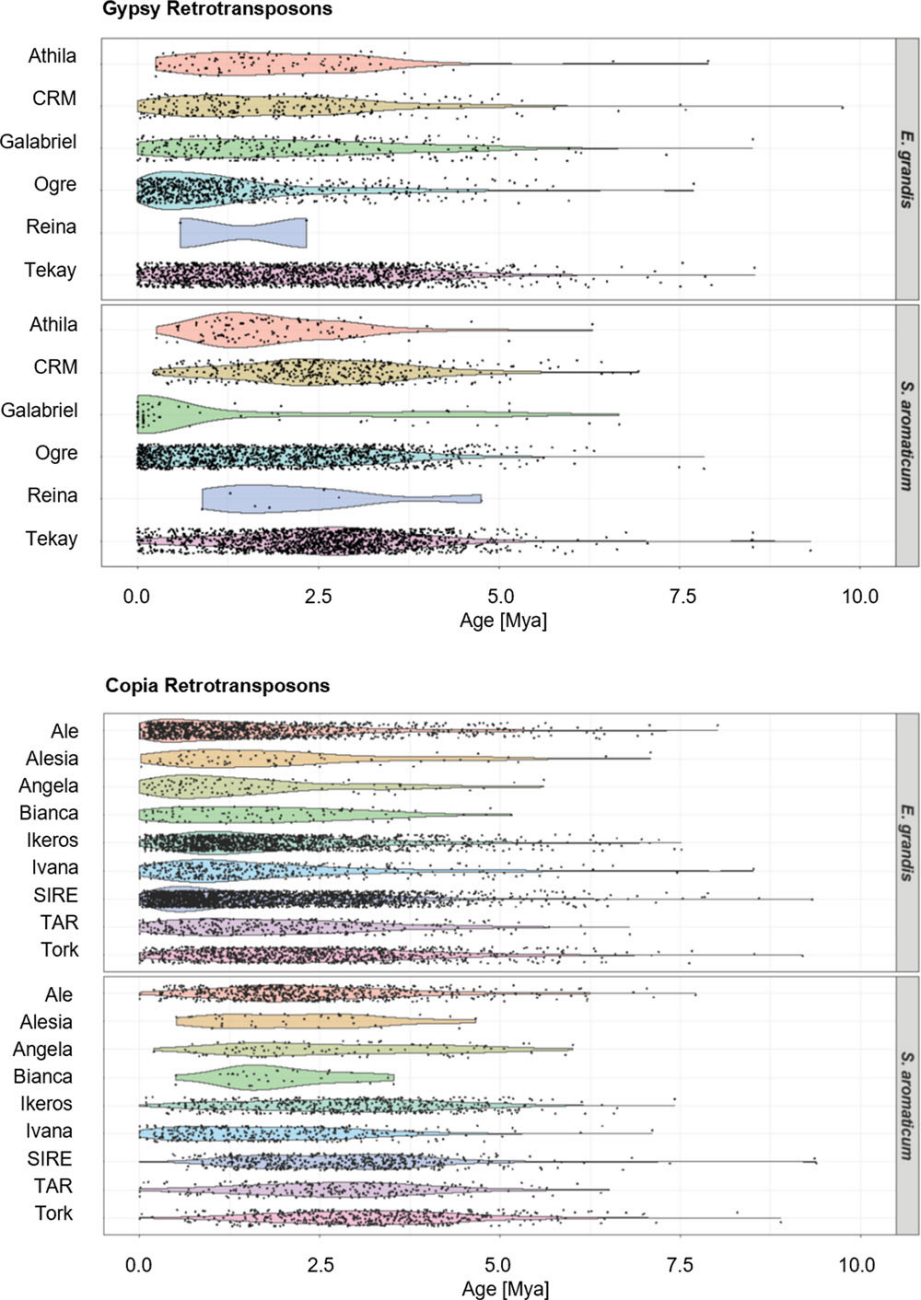
Estimation of LTR-RTs element insertion time. The top panel shows the insertion time of Gypsy lineages in *E. grandis* and *S. aromaticum*, and the bottom panel shows the insertion time of Copia lineages in *E. grandis* and *S. aromaticum*. Only lineages detected in both species are shown.

To further investigate the clove genome evolution, we classified into lineages the LTR retrotransposon (LTR-RTs) elements belonging to the superfamilies Copia and Gypsy in clove and eucalyptus genome assemblies and estimated their insertion timeline (Fig. 2 and Supplementary Tables 3,4). A total of ten Copia lineages (Ale, Bianca, Angela, Ivana, Gymco-IV, Alesia, Ikeros, SIRE, TAR, and Tork) and seven Gypsy lineages (Tekay, Athila, Galadriel, CRM, non-chromo-outgroup, Ogre, and Reina) were identified in clove genome.

Among the 3367 Gypsy elements identified, the lineages Ogre (1244 elements) and Tekay (1538) were the most expanded of the LTR-RTs lineages representing 6.31 and 5.41% of the genome assembly, respectively. The Copia superfamily is less abundant in the clove genome and comprised 2811 elements. The most abundant Copia elements in the clove genome belonged to the lineages SIRE (502 elements), Ale (609), Ikeros (486), Tork (475), Ivana (323), and TAR (241) and represented 1.65, 1.62, 1.46, 1.54, 0.78, and 0.57% of the genome assembly, respectively.

In contrast, we found a higher genomic abundance of Copia (7736 elements) versus Gypsy (2910) LTR-RTs in the eucalyptus genome assembly. The most abundant Copia elements identified in eucalyptus genome assembly, like for clove, belonged to the lineage SIRE (2646 elements), Ale (1302), Ikeros (1660), Tork (1004), Ivana (428), and TAR (369). By estimating the time of insertion of the Copia elements, we observed a higher number of recent insertions of the Copia lineages in the eucalyptus genome (from ~2.5 Mya) when compared to clove (Fig. 2).

We also noticed a distinct dynamic of Gypsy retrotransposons between the two species, especially for the Ogre elements that are more abundant in the clove genome (1244 elements) than in the eucalyptus genome assembly (719). In the clove genome, Ogre elements were inserted into the genome between 0 and ~5 Mya. In eucalyptus, we estimated that most of the Ogre elements were inserted later between 0 and ~2.5 Mya.

### Identification of gene families involved in the biosynthesis of eugenol

**Fig. 3:**
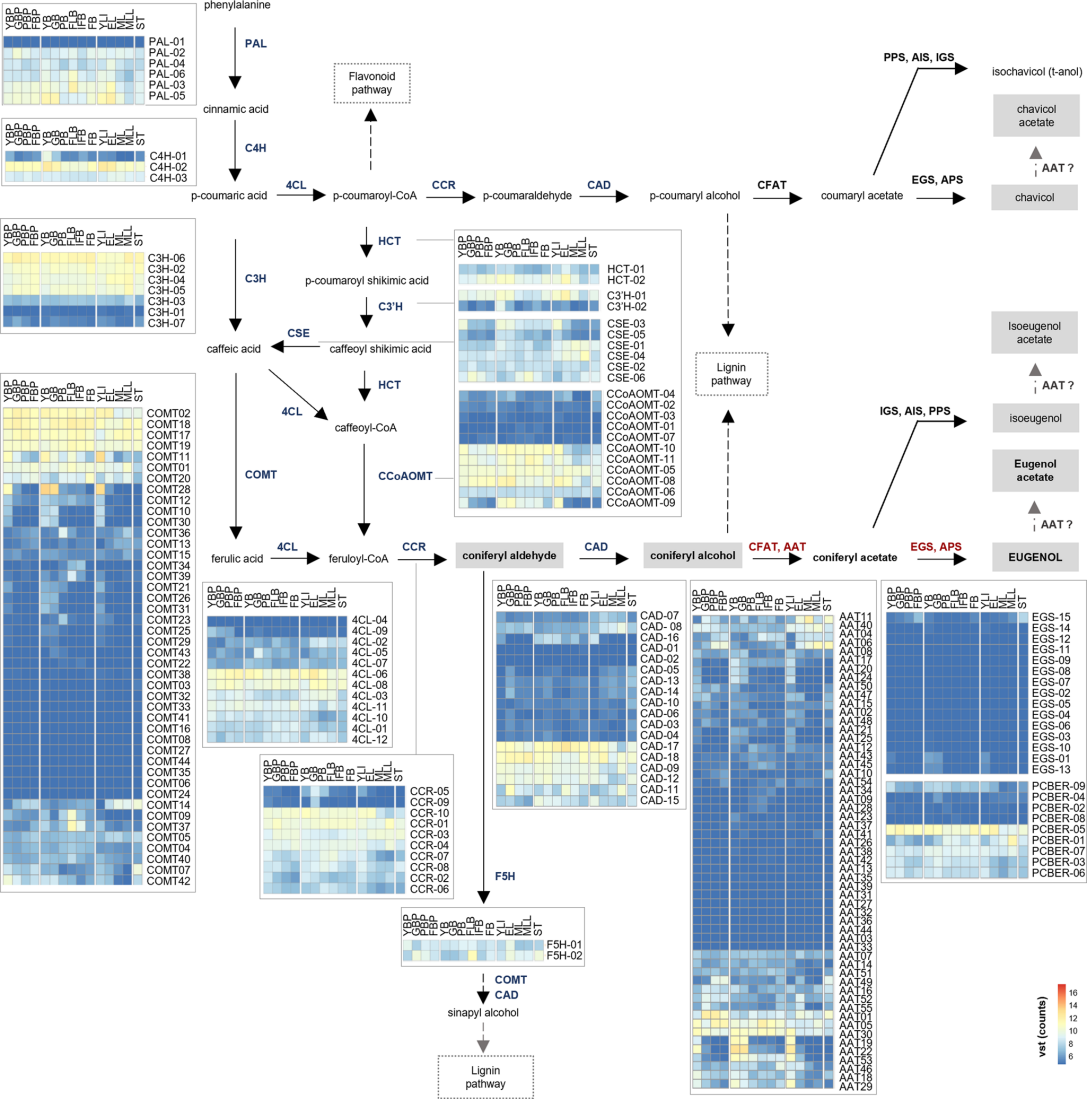
Proposed biosynthetic pathways for lignin precursors and phenylpropene volatiles in *Syzygium aromaticum* (adapted from ref. ^24^ and ref. ^25^) and RNAseq evidence of expression of the identified biosynthetic genes. Gene families involved in the biosynthesis of precursors of lignin, monolignol coniferyl alcohol, coumaryl alcohol, and sinapyl alcohol are indicated in blue; gene families involved in the biosynthesis of coniferyl acetate and eugenol are indicated in red, and those involved in the biosynthesis of the phenylpropenes chavicol, isoeugenol, and t-anol are indicated in black. Compounds detected in clove leaves and buds are in gray boxes. The gray arrows indicate hypothetical reactions leading to the formation of phenylpropenes and their respective esters via the activities of alcohol acyltransferase (AAT). Heatmaps represent the relative expression profiles of the biosynthetic genes in peduncles of young buds (YBP), peduncles of green buds (GBP), peduncles of pink buds (PBP), peduncles of buds in the fruiting stage (FBP), young buds (YB), green buds (GB), pink buds (PB), flowering bud (FLB), buds in the initial fruiting stage (IFB), buds in fruiting stage (FB), young leaves (YLI), expanded pale green leaves (EL), mature leaves (ML), mature leaf laminas (MLL), and stems (ST). *n* = 2 for flowering bud (FLB) and peduncles of pink buds (PBP) and *n* = 3 for all other groups. PAL phenylalanine ammonia-lyase, C4H cinnamate 4-hydroxylase; C3H 4-coumarate 3-hydroxylase, COMT, caffeate/5-hydroxyferulate 3-*O*-methyltransferase, F5H, ferulate 5-hydroxylase/coniferaldehyde 5-hydroxylase, 4CL 4-hydroxycinnamate:CoA ligase, HCT 4-hydroxycinnamoyl CoA:shikimate/quinate hydroxycinnamoyltransferase, C3′H 4-coumaroyl shikimate/quinate 3′-hydroxylase, CSE caffeoyl shikimate esterase, CCoAOMT caffeoyl CoA 3-*O*-methyltransferase, CCR cinnamoyl CoA reductase, CAD cinnamyl alcohol dehydrogenase, CFAT coniferyl alcohol acyltransferase, EGS eugenol synthase, APS allyl-phenylpropene synthase, IGS isoeugenol synthase, PPS propenyl-phenylpropene synthase, AIS anol/isoeugenol synthase, AAT alcohol acyltransferase, VST variance stabilizing transformation.

On the basis of functional annotations, clustering by ortholog best hits, and phylogenetic analysis, we identified 116 putative phenylpropanoid biosynthetic genes belonging to the 11 gene families PAL, C4H, 4CL, HCT, C3′H, CSE, COMT, CCoAOMT, F5H, CCR, and CAD in clove genome (Fig. 3 and Supplementary Data 2, 3). A number of genes were lower than in the eucalyptus genome for which 175 genes were reported for these 11 families^10,29^.

The PAL, 4CL, CCoAOMT, and COMT and CAD families were significantly expanded in the eucalyptus genome when compared to the *Arabidopsis*, *Populus*, and *Oryza* genomes^10^. These five families, along with HCT and C3′H families, were also more expanded in eucalyptus when compared to clove, with a notable difference for the COMT and CAD families for which 23 and 28 additional copies are present in the *E. grandis* genome (Supplementary Fig. 4). For the C4H, CSE, and CCR families, we selected a higher number of putative orthologs in the clove genome, with one additional copy for C4H, and CCR, and five additional copies for the CSE family.

Among genes belonging to the BAHD superfamily in the clove genome, we selected 55 genes encoding for putative AATs (alcohol acyltransferase): 10 genes encoding putative AATs that clustered with selected characterized coniferyl alcohol acyltransferases and 45 putative AATs of classes III and V reported to be involved in the biosynthesis of volatiles esters (Fig. 3, Supplementary Data 2, 3, Supplementary Fig. 5, and Supplementary Note)^30–33^.

EGS is a key enzyme for the biosynthesis of eugenol, it catalyzes eugenol production by using coniferyl acetate as a substrate. Using characterized EGS proteins belonging to the PIP family, we mined a total of 24 genes: 15 genes encoding putative EGS and 9 encoding putative phenylcoumaran benzylic ether reductases (PCBER) (Fig. 3, Supplementary Data 2, 3, and Supplementary Note)^25,34^. A Pfam domain search allowed the identification of seven additional genes encoding proteins containing an NmrA domain, which were considered possible PIP members (NmrA 1–7).

The 15 genes encoding putative EGS are split into two loci. The first locus on chromosome 10 comprises fourteen putative EGS genes (EGS1 to EGS14), and a second locus on chromosome 11 is composed of a single EGS copy (EGS15). The percentage sequence similarity among the fourteen putative EGS encoded by the first cluster is high (75.5–100%). The sequence similarity decreases to 55.4% between the putative EGS encoded by the genes located on different chromosomes.

**Fig. 4:**
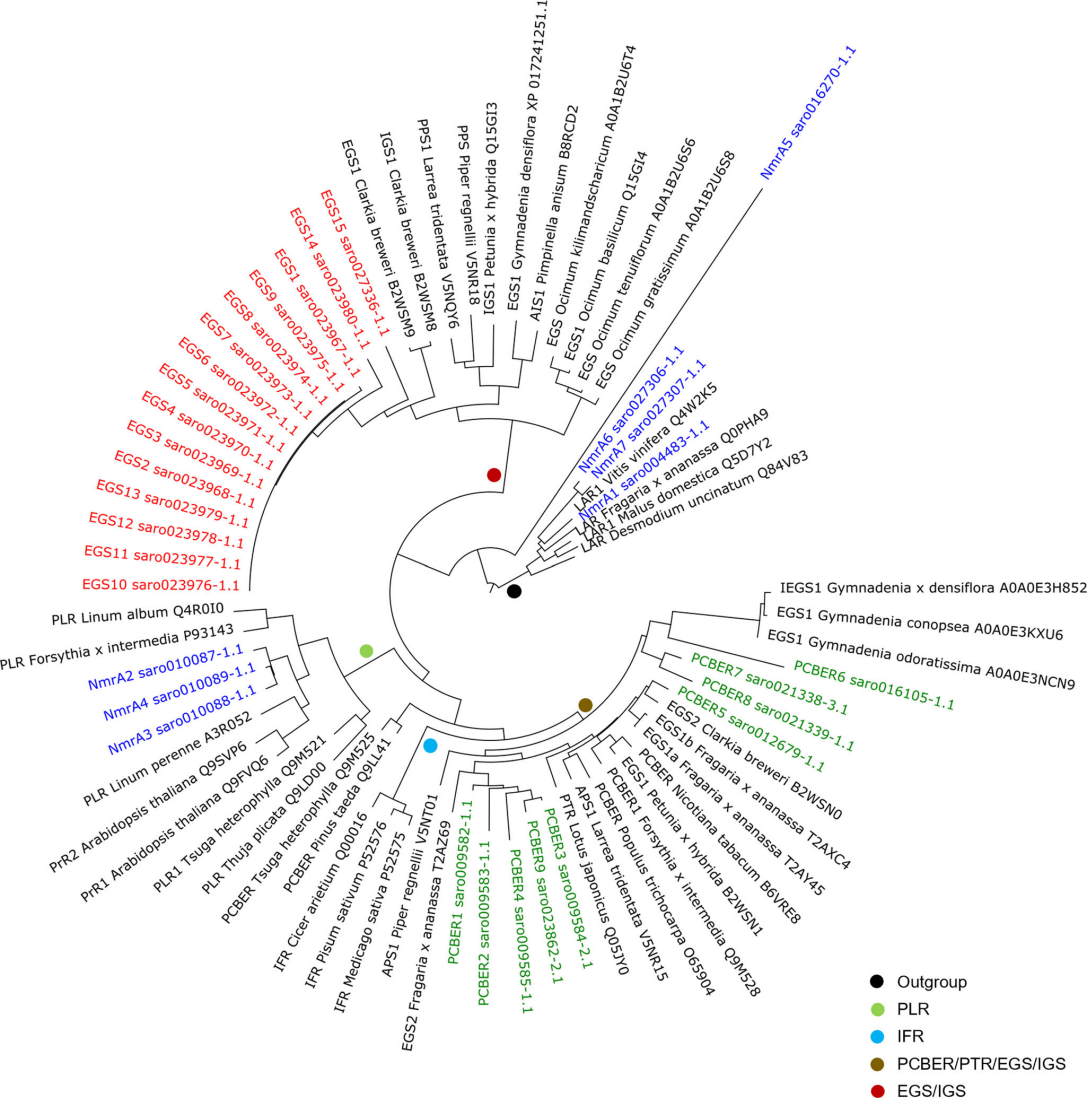
Evolution of selected putative members of the PIP family in *Syzygium aromaticum*. Phylogenetic tree of protein sequences from the PIP family and *S. aromaticum* putative EGS, PCBERs, and proteins containing a NmrA domain possibly involved in the biosynthesis of eugenol. The phylogenetic trees were generated by using Clustal Omega. Groups from Koeduka^25^. EGS eugenol synthase, IGS isoeugenol synthase, AIS anol/isoeugenol synthase, PPS propenyl-phenylpropene synthase, APS allyl-phenylpropene synthase, PTR pterocarpan reductase, PCBER phenylcoumaran benzylic ether reductase, IFR isoflavone reductase, LAR leucoanthocyanidin reductase, PLR pinoresinol–lariciresinol reductase, PrR pinoresinol reductase.

A previous phylogenetic analysis performed with EGS and other members of the PIP family of NADPH-dependent reductases suggested that phenylpropene synthases have independently evolved into two distinct lineages: a group of phenylpropene synthases composed of EGS, IGS, propenyl-phenylpropene synthase (PPS), and allyl-phenylpropene synthase (APS) and the second group of phenylpropene synthases (APS and EGS) that cluster with PCBERs and pterocarpan reductase (PTR)^25^. Our phylogenetic analysis revealed that the 15 putative EGS clustered with the EGS–IGS protein group. Clove putative EGS showed the highest sequence similarity to the *Clarkia breweri* IGS1 and EGS1. The additional nine putative PCBERs clustered with the PCBER/PTR/EGS/IGS group and showed a closer phylogenetic relationship with *Gymnadenia conopsea* EGS1, *G. odoratissima* EGS1, *G. densiflora* IEGS1, *C. breweri* EGS2, *Fragaria. x ananassa* EGS2 (Fig. 4).

### Biosynthesis of eugenol in clove leaves and buds at different growth phases

**Fig. 5:**
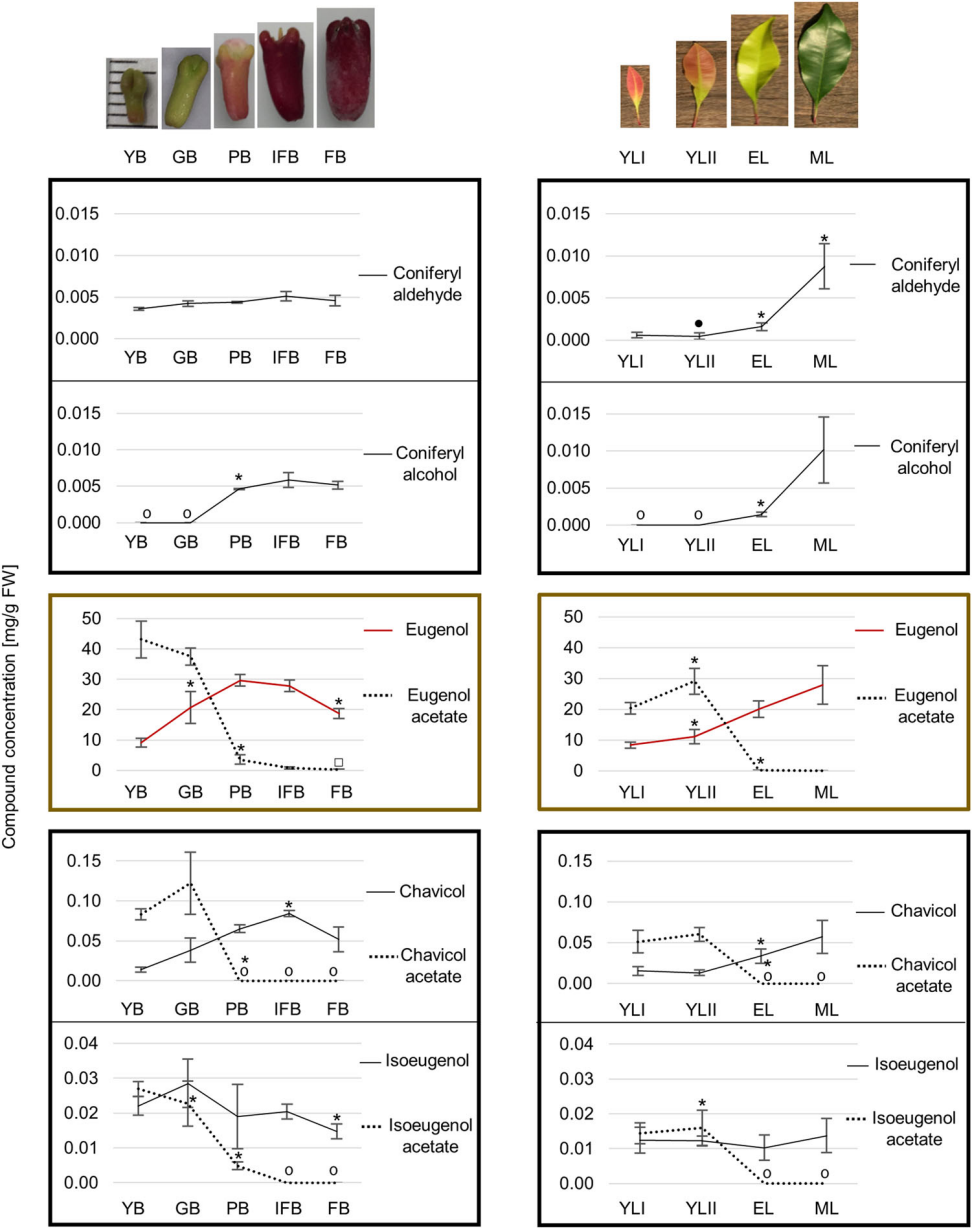
Concentrations (mg/g FW) of the phenylpropenes eugenol, chavicol, and isoeugenol, their respective esters, and the precursors coniferyl aldehyde and coniferyl alcohol in *Syzygium aromaticum* leaves and buds among the growth phases. Data were represented as mean ± SD (*n* = 3). Asterisks indicate significant concentration differences relative to the previous growth phase. Two-tailed *t*-test *P* < 0.05. □ Compound not detected in two of the three organ replicates; ● compound not detected in one of the three organ replicates; O compound not detected. Fresh weight (FW). Young buds (YB), green buds (GB), pink buds (PB), buds in the initial fruiting stage (IFB), buds in fruiting stage (FB), young leaves (YLI), expanded young leaves (YLII), expanded pale green leaves (EL), and mature leaves (ML).

Clove leaves and buds at different growth phases were analysed by UHPLC and GC–MS (Fig. 5). The major components found in clove leaves and buds are the major compounds found in clove EO: the phenylpropenes eugenol (8.3–29.7 mg/g fresh weight [FW]) and eugenol acetate (0.05–43.1 mg/g FW) and the sesquiterpenes β-caryophyllene (3.07–5.6 mg/g FW) and α-humulene (0.2–0.6 mg/g FW) (Supplementary Data 4). The concentration of eugenol increased during the development of clove leaves and buds to reach a maximum in mature leaves (27.9 ± 6.2 mg/g of FW) and full-budding-stage pink buds (29.7 ± 1.9 mg/g of FW), the stage at which they are collected (before the flowering stage). In contrast, the concentrations of eugenol acetate—which had accumulated in large amounts in young leaves and buds—decreased during organ growth, and only traces of the compound were detected in mature leaves and buds at the fruiting stage. In young leaves and buds, not only eugenol but also the phenylpropenes isoeugenol and chavicol (produced in smaller amounts) were found in their ester forms, while, in older leaves and buds, their alcohol forms were predominant. Coniferyl alcohol, the phenylpropanoid precursor of lignin, remained undetected in young leaves and buds. However, in older leaves and buds, the concentrations of coniferyl alcohol were significantly increased. Coumaryl acetate and coniferyl acetate (substrate of EGS) were not detected. The methyl derivative forms of phenylpropenes were not detected in either leaves or buds, despite the small percentage of methyl eugenol that was reported in EO (0.05–0.2%)^18^.

**Fig. 6:**
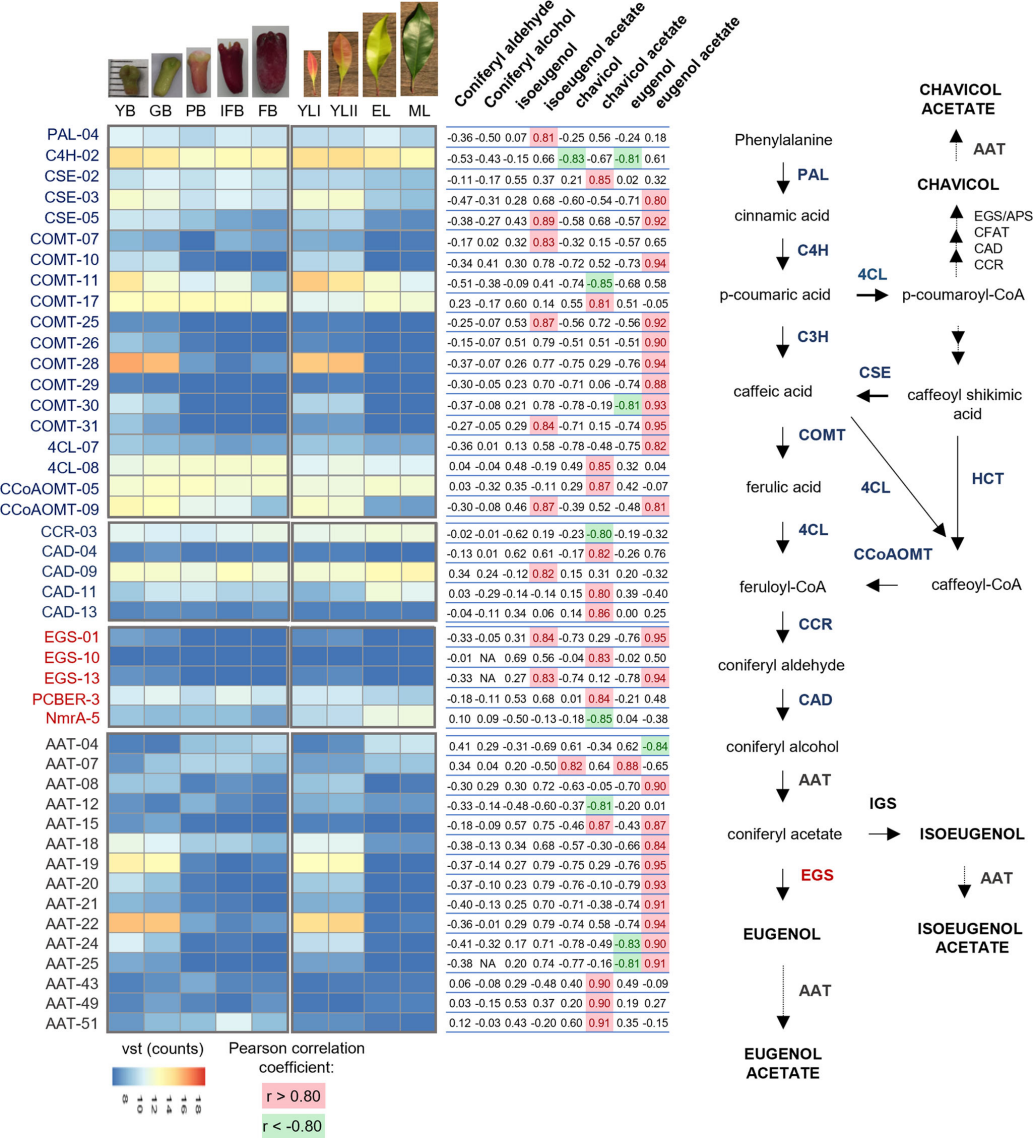
List of genes possibly involved in the biosynthesis of eugenol and eugenol acetate in leaves and buds at different growth phases according to the combined transcriptomic and metabolomic data. Heatmaps indicate the expression profiles of genes correlated with the concentrations of the phenylpropenes eugenol and chavicol, their respective esters, isoeugenol acetate, and the precursor coniferyl alcohol (Pearson correlation coefficient *r* > 0.8 to *r* < −0.8, *n* = 3). Young buds (YB), green buds (GB), pink buds (PB), buds in the initial fruiting stage (IFB), buds in fruiting stage (FB), young leaves (YLI), expanded young leaves (YLII), expanded pale green leaves (EL) and mature leaves (ML). PAL phenylalanine ammonia-lyase, C4H cinnamate 4-hydroxylase, C3H 4-coumarate 3-hydroxylase, COMT caffeate/5-hydroxyferulate 3-*O*-methyltransferase, 4CL 4-hydroxycinnamate:CoA ligase, HCT 4-hydroxycinnamoyl CoA:shikimate/quinate hydroxycinnamoyltransferase, CSE caffeoyl shikimate esterase, CCoAOMT caffeoyl CoA 3-O-methyltransferase, CCR cinnamoyl CoA reductase, CAD cinnamyl alcohol dehydrogenase, CFAT coniferyl alcohol acyltransferase, EGS eugenol synthase, APS allyl-phenylpropene synthase, IGS isoeugenol synthase, AAT alcohol acyltransferase, VST variance stabilizing transformation.

**Fig. 7:**
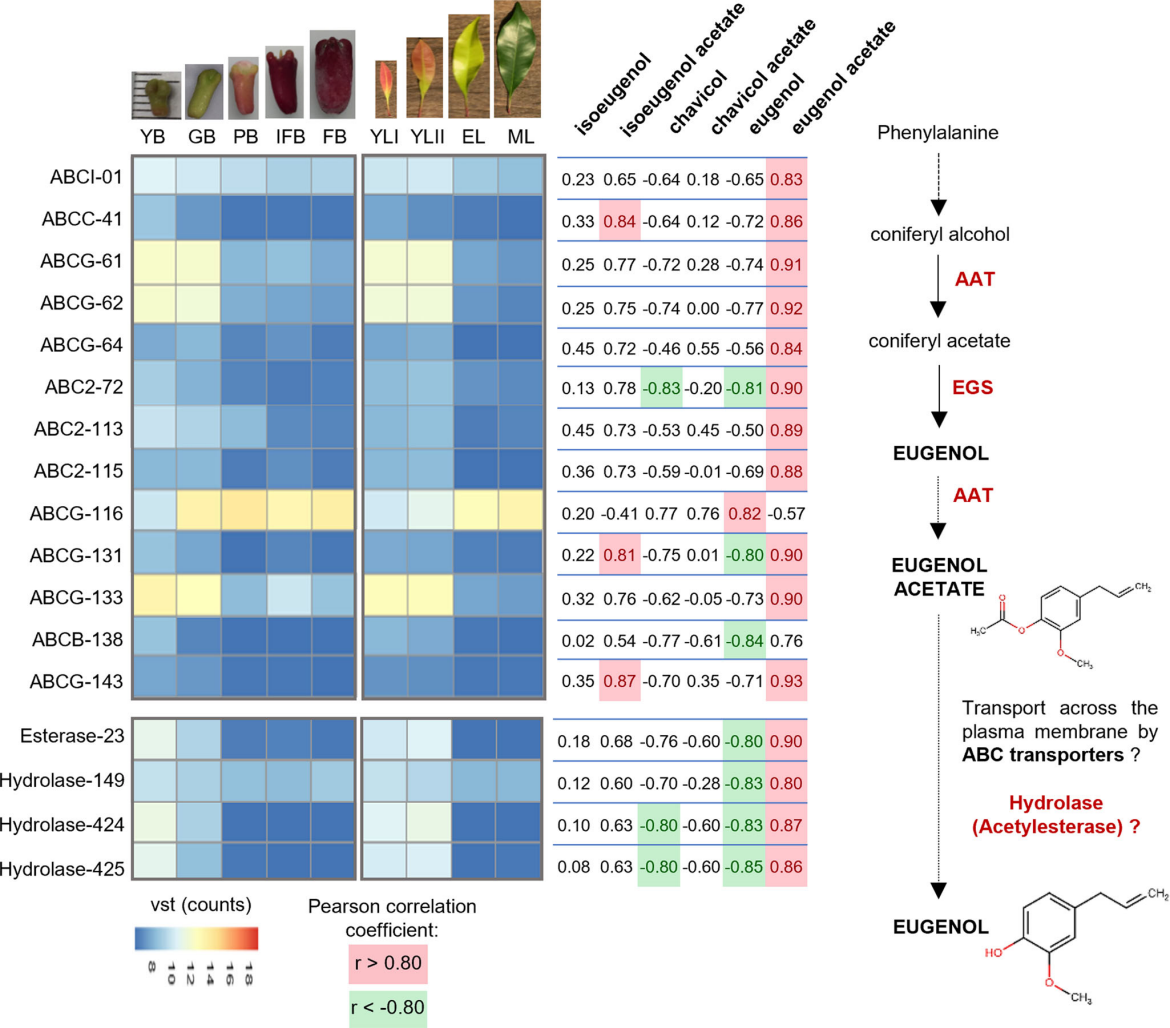
Biosynthesis of eugenol in *Syzygium aromaticum* leaves and buds. Proposed hypothetical scenario in which eugenol acetate is converted to eugenol, on the basis of transcriptomic and metabolomic data analysis. Heatmaps indicate the expression profiles of genes correlated with the concentrations of the phenylpropenes eugenol, eugenol acetate, chavicol, and isoeugenol acetate (Pearson correlation coefficient *r* > 0.8 to *r* < −0.8, *n* = 3). AAT alcohol acyltransferase, EGS eugenol synthase, ABC ATP-binding cassette transporter. Young buds (YB), green buds (GB), pink buds (PB), buds in the initial fruiting stage (IFB), buds in fruiting stage (FB), young leaves (YLI), expanded young leaves (YLII), expanded pale green leaves (EL), and mature leaves (ML). VST variance stabilizing transformation.

Twenty-four genes identified as phenylpropanoid genes from eight families (PAL, C4H, CSE, COMT, 4CL, CCoAOMT, CCR, and CAD) were found to be correlated with the concentration of the phenylpropenes isoeugenol, chavicol, and eugenol and their ester forms. These included eleven genes that were especially positively correlated with the concentration of eugenol acetate in clove leaves and buds (Fig. 6). Among the members of the PIP family, the gene expression levels of two putative EGS (EGS1 and EGS13) were positively correlated with the concentration of eugenol acetate. The expression levels of these two genes were higher in young leaves and buds than in the mature stages, where the concentration of eugenol was increased (Fig. 6). The biosynthesis of eugenol via conversion of coniferyl acetate into eugenol by EGS does seem to explain the increase in eugenol concentration in mature leaves and buds, as the expression levels of the EGS genes were lower in mature organs. The expression of nine genes encoding putative AATs (AAT-8, AAT-15, AAT-18 to AAT-22, AAT-24, and AAT-25) was positively correlated with eugenol acetate concentration and for two genes also negatively correlated with eugenol concentration (AAT-24 and AAT-25). Similar to the results observed for the EGS protein genes, the expression levels of these nine AAT genes in young leaves and buds were higher than those in the mature stages. In this study, eugenol acetate is the main compound detected in young leaves and buds, and this large concentration of eugenol acetate is probably not completely volatilized or degraded during organ growth. However, the enzymatic hydrolysis of eugenol acetate produced in young leaves and buds to eugenol might explain the increase in eugenol levels in mature buds and leaves despite the lower expression levels of EGS genes. We identified four predicted proteins annotated as hydrolase (hydrolase-149, 424, and 425) and esterase (esterase-23) that were positively correlated with the concentration of eugenol acetate and negatively correlated with the concentration of eugenol (Fig. 7). The expression levels of these four genes in young leaves and buds were higher than those in the mature stages, suggesting that these genes could be involved in the hydrolysis of eugenol acetate into eugenol during the growth of both organs. ATP-binding cassette (ABC) transporters constitute a ubiquitous superfamily of proteins involved in the transport of plant secondary metabolites^35,36^. We found eleven genes encoding putative ABC transporters that were positively correlated with eugenol acetate. Analysis of the expression levels of these ABC genes indicated that ten of them (ABCI-1, ABCC-41, ABCG-61, ABCG-62, ABCG-64, ABC2-72, ABC2-113, ABC2-115, ABCG-131, and ABCG-143) were more highly expressed in young organs than in mature organs, suggesting that they might be involved in the transport of the eugenol acetate present in large quantities only in young leaves and buds. In contrast, one ABC transporter (ABCG-116) was positively correlated with eugenol concentration and more highly expressed in mature leaves and buds, and might be involved in the transport of the eugenol (Fig. 7) (Supplementary Data 3).

## Discussion

The current Myrtaceae infrafamilial classification recognizes seventeen tribes^37^. The *Eucalyptus* and *Corymbia* sister genera belong to the tribe Eucalypteae, and clove belongs to the tribe Syzygieae. The Eucalypteae tribe diverged from other Myrtaceae ~52–59 Mya^38,39^. The estimated crown age of the Syzygieae tribe indicates that the tribe originated later, at ~22.5–36.4 Mya^39,40^. An intergeneric comparison study using a high-density linkage map for the *C. citriodora* subsp. *variegata* (370 Mb) and *E. grandis* (640 Mb) genomes revealed that the overall genome structure was conserved among these eucalypt species and reported the presence of intrachromosomal rearrangements on seven of the 11 chromosomes (2, 4, 6, 8, 9, 10, and 11). However, the rearrangements located on two linkage groups, 2 and 8, of *C. citriodora* subsp. *variegata* could not be validated^28^. Our inter-tribe comparative genomic analysis between *S. aromaticum* (370 Mb) and *E. grandis* revealed that the genome architecture was also largely conserved between the two Myrtaceae species. By comparing our results with the rearrangements previously reported between eucalyptus and *C. citriodora* subsp. *variegata*, we observed that the same seven chromosomes (2, 4, 6, 8, 9, 10, and 11) were impacted by intrachromosomal rearrangements in clove and *C. citriodora* subsp. *variegata*, suggesting that these seven chromosomes were more subjected to rearrangements and may have played a key role in diversification of the tribes and genera in this family. The presence of similar large terminal inversion on chromosomes 4 (c), 9 (h), 10 (i), and 11 (j) of clove and the corresponding linkage groups of *C. citriodora* subsp. *variegata* suggests that these rearrangements may have occurred in the *Eucalyptus* genus. In contrast, *Syzygium* and *Corymbia* seem to have retained the chromosome structure of a common ancestor to the two tribes Syzygieae and Eucalypteae, which split ~65–70 Mya in the late Cretaceous, during the separation of the clades Syncarpieae, Eucalypteae, Leptospermeae, Chamelaucieae, and Lindsayomyrteae from the clades Backhousieae, Syzygieae, Tristanieae, Metrosidereae, Myrteae, and Kanieae^39^. Additional large terminal inversions, not reported for *C. citriodora* subsp. *variegata*, occurred in *S. aromaticum*, revealing the continued evolution of the genome architecture in the Syzygieae tribe, possibly specific to genome speciation and the evolutionary process of the *Syzygium* genus.

Inversions are chromosomal rearrangements which may play a prominent role in local adaptation and speciation^41^. Nevertheless, inversions may also be the consequences of sequencing and assembly artefacts. The absence of Hi-C data to scaffold a genome and the low marker density in regions of high-density linkage maps could for instance lead to the observations of inversions or rearrangements in comparative genomic studies. The hypothetical evolutionary scenarios of the chromosome structure presented in this study implies that the inversions observed resulted from evolutionary events. Although our scaffolding results are well supported by the Hi-C data, new comparative genomic studies with additional chromosome-scale genomes from *Syzygium* species are needed to better understand the evolutionary history of the chromosomal rearrangements that occurred in the *S. aromaticum* genome and to gain insights into the impact of the rearrangements on the evolution of the Syzygieae tribe and its actual diversity.

Transposable elements (TEs) are mobile genomic DNA that contributes to the evolution and function of genomes^42–44^. Among TEs identified in the clove genome, the long terminal repeat retrotransposons (LTR-RTs) were the most abundant representing 21.8% of the genome assembly, a fraction similar to that reported for eucalyptus (21.9%)^10^. Most of the LTR-RTs found in the clove genome belonged to Gypsy superfamily as it was reported for *Populus* and the relative Myrtales species pomegranate^45,46^. The fraction of Gypsy (13.2%) and Copia (8.1%) retrotransposons identified in the clove genome were consistent with those reported for the pomegranate 274 Mb genome assembly (Gypsy (11.55%) and Copia (5.87%)). In contrast, we found a higher genomic abundance of Copia versus Gypsy LT- RTs in eucalyptus assembly (690 Mb). The differential accumulation of the Copia and the Gypsy lineages in the clove and eucalyptus genome assemblies indicated that a notable distinct activity of both Gypsy and Copia superfamilies occurred in the two Myrtaceae species genomes. This first analysis of clove LTR-RTs Gypsy and Copia repertories provides a valuable resource for future comparative analysis aiming to infer the impact of specific LTR-RTs lineages or elements on the clove genome architecture, speciation, and biology.

The chemical composition of clove EO has been extensively investigated and found to be influenced by the geographical origin and age of the tree, the organ and its stage of development, and postharvest processing (e.g., drying and storage)^18,47–51^. It was also reported that clove buds contain a large amount of eugenol, with a eugenol concentration of 9381.7 mg/100 g dry weight being reported for dried clove—a concentration 49 times higher than that in sweet basil (189.6 mg/100 g dry weight)^52^. In this study, we performed a combined transcriptomics and metabolomics experiment using the leaves and buds of clove at different growth phases to investigate the genomic basis of the biosynthesis of eugenol. Our results suggest that the lower concentration of eugenol found in young compared to mature buds and leaves might be the result of the conversion by AATs of eugenol into eugenol acetate. This conversion might be reversed by the hydrolysis of eugenol acetate into eugenol during the growth of both organs. On the basis of our correlation analysis, we hypothesized that eugenol acetate might be actively transported by ABC transporters across the plasma membrane of the cells occurred before being hydrolyzed into eugenol.

Genes involved in the biosynthesis of eugenol belong to large families, which are not restricted to the biosynthesis of lignin precursors or production of eugenol^23,25,30^. The identification of genes family was performed using a homology-based approach. This approach relies on multiple factors affecting its efficacy such as the accuracy of the clove assembly sequences, the transcript and protein predictions, the selected reference proteins, and the identity and coverage cut-off used to select a number of best hits or genes of interest. The list of selected genes potentially involved in the biosynthesis of eugenol was narrowed on the basis of correlation (Pearson correlation coefficient *r* ≥ 0.8 or *r* ≤ −0.8) between expression pattern and eugenol and eugenol acetate concentration. In this study, we did neither validate the functional prediction of the genes selected nor their role in the biosynthesis of eugenol. Nevertheless, our combined transcriptomic and metabolomic data provide a first insight into the genetic basis of eugenol biosynthesis in clove leaves and buds. In addition, the list of selected gene candidates represents a starting point for further characterization of the putative biosynthetic enzymes. Eugenol is an important compound that affects the value of clove products and a biocompound with a high potential for human health benefits^21,53–56^. Additional studies on different organs (stems, peduncles, and roots) and specific anatomical structures (e.g., secretory cavities) will be required to further investigate the high eugenol accumulation in EO and its emission by the bud^57^.

## Methods

### Biological material

The clove reference genome was generated from a single clove tree which originated in Masoala National Park in Madagascar and has been growing in Masoala Hall of the Zurich Zoo in Switzerland since 2007 under controlled climatic conditions (Collection number JA 318 A). The air temperature is maintained between 20 and 30 °C, and the relative humidity is ~80% with up to 6 mm of tropical rainfall per day to replicate the climatic conditions of the Masoala Peninsula of Madagascar. Clove leaves, stems, buds, and peduncles collected from this tree were stored at −80 °C until metabolite and nucleic acid extraction.

### ONT sequencing library preparation and sequencing

High-molecular-weight DNA was isolated from frozen leaves by using the “ONT high-molecular-weight gDNA extraction from plant leaves” protocol. Sequencing libraries were prepared for sequencing on minION flow cells (FLOW-MIN106D R9 version) by using ligation sequencing (SQK-LSK109) and flow cell priming (EXP-FLP001) kits (Oxford Nanopore Technologies, Oxford, UK). Base-calling was performed by using Guppy 4.0.15 in the high-accuracy mode. Raw ONT reads were cleaned by using SeqKit^58^. Reads shorter than 10000 bp or with a quality score lower than 7 were discarded.

### DNAseq library preparation and Illumina sequencing

DNAseq libraries were generated from total DNA isolated from frozen leaves by using the “ONT high-molecular-weight gDNA extraction from plant leaves” protocol (Oxford Nanopore Technologies, Oxford, UK) and prepared using the Celero PCR workflow with an enzymatic fragmentation kit from Nugen (San Carlos, CA, USA). DNAseq libraries were then loaded on an Illumina flow cell S2 and sequenced on the Illumina Novaseq6000 instrument (San Diego, CA, USA) as 2 × 151 bp PE reads. Raw reads were cleaned by using fastp to remove low complexity sequences and adapter sequences^59^.

### Hi-C library preparation and Illumina sequencing

Hi-C libraries were prepared from 0.2 g of frozen leaves by using the Proximo Hi-C Kit in accordance with the manufacturer’s instructions (Phase Genomics, Seattle, WA). The libraries were then sequenced with an Illumina HiSeq 2500 instrument (San Diego, CA, USA) as 2 × 101 bp PE reads. Raw reads were cleaned by using fastp^59^.

### Estimation of genome size and percentage heterozygosity

Cleaned Illumina PE reads from DNAseq libraries were analyzed by GenomeScope 2.0 to estimate genome size and percentage heterozygosity by using a k-mer size equal to 21 bp^26^.

### De novo genome assembly

In order to reduce the raw error rate, cleaned ONT reads were first corrected with Ratatosk^60^ by using the cleaned Illumina PE short reads from DNAseq libraries. Ratatosk corrected ONT reads were then mapped by minimap2^61^ to the eucalyptus chloroplast and mitochondrial genomes to remove reads originating from plastid genomes. The filtered and corrected ONT reads were assembled by using minimap2^61^ and miniasm^62^. The resulting raw assembly was first polished with minipolish^63^ by using corrected ONT reads, and then with ntEdit^64^ by using Illumina PE short reads from DNAseq libraries. Haplotigs were detected and removed by using Purge_dups^65^.

Cleaned Illumina Hi-C reads were mapped to the haplotig-purged primary contigs by using BWA-MEM^66^. The scaffolding to a chromosome-level assembly was performed by using YAHS (https://github.com/c-zhou/yahs). Hi-C map files were generated with PretextMap (https://github.com/wtsi-hpag/PretextMap) and used to manually curate the assembly using PretextView (https://github.com/wtsi-hpag/PretextView). Finally, the curated clove genome was mapped to the *E. grandis* genome (https://eucgenie.org) by using minimap2^61^, visualized by using a customized R script, and the orientation and names of the clove chromosomes set in accordance with those of *E. grandis*. The completeness of the final chromosome-level assembly was assessed by using the genome evaluation mode of BUSCO (version 5.2.2) and the eudicots_odb10 lineage dataset^27^. The quality of the final assembly was estimated using Yak^67^.

### RNAseq library preparation for Illumina sequencing

Total RNA was isolated in sextuplicate from whole leaves (young red leaves, expanded pale green leaves, and dark green mature leaves) and buds (young buds, green buds, pink buds, buds in the initial fruiting stage, and buds in the fruiting stage); in triplicate from the stem, expanded young leaves, lamina of mature leaves, peduncles (of young buds, green buds, pink buds, buds in the fruiting stage); and in duplicate from flowers and peduncles of pink and flowering buds.

Total RNA was extracted from frozen powder by using Ambion PureLink Plant RNA Reagent (Ambion by Life Technologies; catalog number 12322-012) in accordance with the manufacturer’s protocol (manual MAN0000243, revision 2.0). The concentration and quality of total RNA were assessed with an Agilent Bioanalyzer by using the Agilent RNA 6000 Nano Kit. mRNA libraries were prepared from 500 ng of total RNA by using the Nugen Universal Plus mRNA-Seq library preparation kit with NuQuant^®^ (San Carlos, CA, USA). Quality control was performed by using an Agilent Bioanalyzer and Agilent DNA 1000 Kit. The libraries were sequenced on a HiSeq 4000 instrument as 2 × 151 bp PE reads.

### Gene model prediction and functional annotation

Illumina RNAseq reads were cleaned and overlapping paired reads merged using fastp before being mapped to the assembly by using minimap2. Gene models were created by sample using Scallop^68^. Similarly, *E. grandis* transcripts were mapped to the assembly by using minimap2, and gene models were created by using bedtools bamtobed^69^.

Augustus^70^ was trained using Complete BUSCO genes from the eudicot_odb10 and used to predict ab initio and evidence-based genes hinted by the RNAseq and *E. grandis* gene models. The final gene models were created by merging the RNAseq and *E. grandis* gene models with evidence-based and ab initio Augustus predictions using taco^71^ and adding CDS using Transdecoder (https://github.com/TransDecoder/TransDecoder/wiki) and gffread^72^. The Malvids portion of UniRef was used to annotate the gene models with their best hit by using DIAMOND^73^. Pfam^74^ domains were annotated by using HMMER^75^.

### Prediction and annotation of repeat elements

TEnester^76^ and TEsorter^77^ with REXdb^78^ were used to predict and annotate transposable elements and their insertion age was calculated as previously reported^79^. In addition, repeats were predicted using Red^80^, MITEs using GRF^81^, helitron using EAHelitron^82^, and tandem repeats using tantan^83^.

### Synteny between *S*. *aromaticum* and *E*. *grandis*

Identification of putative homologous chromosomal regions was performed with MCScanX^84^ by using pairwise alignments of the predicted proteins, and a syntenic block size of at least five collinear protein pairs was visualized by using SynVisio (https://synvisio.github.io/#/).

### Identification of gene families involved in eugenol biosynthesis

To identify the genes encoding putative proteins involved in the biosynthesis of eugenol, the full set of clove transcripts was compared with proteins of eucalyptus (PAL, C4H, 4CL, C3′H, HCT, CSE, COMT, CCoAOMT, and F5H) and selected C3H, AAT, and EGS sequences (characterized protein sequences from UniProt) (Supplementary Data 2). The best hits were selected, and phylogenetic analysis of the selected putative proteins and characterized proteins from UniProt was also performed to filter the selected genes.

### Sequences and phylogenetic analysis

Multiple alignment of protein sequences was performed by using CLUSTAL OMEGA^85^. A phylogenetic tree of the PIP family protein sequences and *S. aromaticum* EGS sequences was generated by using FastTree^86^, newick_utils^87^, and ete3^88^.

### Combined metabolome and transcriptome analysis

Total RNA and metabolites were extracted from leaves at four different growth phases: young leaves (5.1–6.2 cm), expanded young leaves (8.7–9 cm), expanded pale green leaves (10.8–12.5 cm), and dark green mature leaves (14.7–15 cm).

Total RNA and metabolites were extracted from buds at five different growth phases: pooled young buds (<0.5 cm), green buds (1.3–1.4 cm), pink buds (full-budding stage; 1.7–1.9 cm), buds at the initial fruiting stage (1.5–1.9 cm), and buds at the fruiting stage (1.8–1.9 cm).

Leaves and buds were sampled in triplicate for each growth phase and stored at −80 °C before being ground in liquid nitrogen. The ground material obtained with each sample was aliquoted into three tubes and weighed to proceed separately to RNA isolation, eugenol and eugenol acetate extraction, and volatile compound extraction.

Total RNA extraction was performed by using Ambion PureLink Plant RNA Reagent (Ambion by Life Technologies; catalog number 12322-012) in accordance with the manufacturer’s protocol (manual MAN0000243, revision 2.0).

Eugenol and eugenol acetate were extracted from frozen leaf and bud powder by adding 10 volumes of methanol (w/v). The mixture of powder and methanol was then placed in a Qiagen TissueLyser II for 3 min at 30 Hz. The samples were then centrifuged for 2 min at 14,800 rpm. The supernatant was diluted 1:10 with methanol for analysis by ultra-high performance liquid chromatography in ultraviolet mode (UHPLC-UV) on a Waters Acquity^TM^ UPLC instrument at 280 and 272 nm for eugenol and eugenol acetate, respectively. Calibration curves built from eugenol and eugenol acetate standard solutions from 2 to 200 µg/mL were used for quantification.

Volatile compounds were extracted from frozen leaf and bud powder by adding 10 volumes of dichloromethane (w/v). The mixture of powder and dichloromethane was then placed in a Qiagen TissueLyser II for 2 min at 30 Hz, with a 30-s pause every 30 s. The samples were centrifuged for 2 min at 14,800 rpm, and the supernatant was filtered. The volatile compound extracts were analyzed in undiluted form and diluted (1:10 in dichloromethane) form. Gas chromatography–mass spectrometry (GC–MS) analysis was performed on an Agilent 7890B GC system equipped with a 5977 A mass selective detector and an OPTIMA 5 MS Accent separation column (30 m × 0.25 mm; 0.25 µm [Macherey Nagel, Germany]). The samples were injected in the splitless mode and at an inlet temperature of 270 °C. The following GC–MS conditions were used for analysis: MS source, 230 °C; MS Quad, 150 °C; transfer line, 280 °C; oven program, 60 °C for 7 min (10 °C/min until 120 °C, and then 5 °C/min until 280 °C); and run time, 45 min. Volatile compounds were identified by comparison of spectra against the database NIST MS search 2.0 and quantified by using standard peak areas (methyl chavicol, caryophyllene, caryophyllene oxide, and β-farnesene) and calibration curves.

Statistical analysis of the metabolomic results was performed by unpaired, two-tailed *t*-tests. *P* < 0.05 was considered significant.

### Differential gene expression profiles between clove tissues

Gene expression analysis was performed with DESeq2^89^ by using the gene-level count matrix obtained from STAR^90^. The variance stabilizing transformation was used before generating heatmaps with pheatmap^91^.

### Reporting summary

Further information on research design is available in the Nature Research Reporting Summary linked to this article.

## Supplementary information

Supplementary Information

Description of Additional Supplementary Files

Supplementary Data 1

Supplementary Data 2

Supplementary Data 3

Supplementary Data 4

Reporting Summary

## Data Availability

Illumina and Oxford Nanopore reads are available from the National Center for Biotechnology Information Short Read Archive (SRA) under accession: PRJNA660399. This Whole Genome Shotgun project has been deposited at DDBJ/ENA/GenBank under the accession JACTMA000000000. The version described in this paper is version JACTMA0100000000. The genome annotation is available from Zenodo at 10.5281/zenodo.6579856. All additional data were available from the corresponding author (Nikolai V. Ivanov) on reasonable request.
