# Author Correction: The clove (*Syzygium aromaticum*) genome provides insights into the eugenol biosynthesis pathway

**DOI:** 10.1038/s42003-025-07487-0

**Published:** 2025-01-21

**Authors:** Sonia Ouadi, Nicolas Sierro, Simon Goepfert, Lucien Bovet, Gaetan Glauser, Armelle Vallat, Manuel C. Peitsch, Felix Kessler, Nikolai V. Ivanov

**Affiliations:** 1 Faculty of Sciences, Laboratory of Plant Physiology, University of Neuchâtel, 2000 Neuchâtel, Switzerland; 2 PMI R&D, Philip Morris Products S. A, Quai Jeanrenaud 5, CH-2000 Neuchâtel, Switzerland; 3 Faculty of Sciences, Neuchâtel Platform of Analytical Chemistry, University of Neuchâtel, 2000 Neuchâtel, Switzerland

Correction to: *Communications Biology* 10.1038/s42003-022-03618-z, published online 09 July 2022

In this article the funding from ‘Philip Morris International is the sole source of funding and sponsor of this research’ was omitted. The original article has been corrected.

