# Supplementary material for: The clove (Syzygium aromaticum) genome provides insights into the eugenol biosynthesis pathway
Source: Commun Biol. 2022 Jul 9;5:684. doi: 10.1038/s42003-022-03618-z (PMC9271057; doi:10.1038/s42003-022-03618-z)
Supplement: Supplementary file 1 — Supplementary Information [file 42003_2022_3618_MOESM1_ESM.pdf]

Supplementary Table 1. Statistics of clove genome assembly process

	**Polished contigs**	**Primary contigs** (Haplotig-purged)	**Final assembly** (Chromosome-level)
Number of sequences	415	202	24
Minimum length (bp)	43,250	10,129	11,734
Maximum length (bp)	10,157,946	10,157,946	43,763,418
Average length (bp)	1,004,870.81	1,832,780.95	15,427,406.3
N	0	0	36,000
%N	0.00	0.00	0.01%
N50	3,352,581	3,818,138	35,418,074
I50	36	30	5
Total length (bp)	417,021,388	370,221,752	370,257,752

Supplementary Table 2. Genomics coordinates of the main rearrangements (a to j) detected between the chromosomes of *S. aromaticum* and *E. grandis.*

Species	Chromosome	Chr length (bp)	Rearrangement	Start (Mbp)	End (Mbp)
S. aromaticum	Chr02	36,857,792	a	22.3	25.7
	Chr02	36,857,792	a	36.1	36.5
	Chr04	27,127,432	b	1.2	8.5
	Chr04	27,127,432	c	18.2	25.6
	Chr06	42,993,409	d	0.0	9.7
	Chr06	42,993,409	e	23.7	28.3
	Chr06	42,993,409	e	36.9	41.5
	Chr08	43,763,418	f	2.4	2.5
	Chr08	43,763,418	f	14.5	21.9
	Chr09	23,380,519	g	1.1	8.3
	Chr09	23,380,519	h	11.3	20.7
	Chr10	26,565,453	i	2.4	11.3
	Chr11	31,862,996	j	22.1	31.8
E. grandis	Chr02	59,529,170	a	55.6	59.1
	Chr02	59,529,170	a	41.4	41.9
	Chr04	41,160,059	b	8.1	21.2
	Chr04	41,160,059	c	29.0	38.9
	Chr06	57,472,304	d	2.2	20.0
	Chr06	57,472,304	e	49.5	56.3
	Chr06	57,472,304	e	36.9	41.9
	Chr08	72,515,979	f	39.4	39.5
	Chr08	72,515,979	f	2.8	12.5
	Chr09	39,307,835	g	0.1	15.1
	Chr09	39,307,835	h	21.6	35.1
	Chr10	37,777,128	i	1.3	12.6
	Chr11	44,836,791	j	29.8	44.6

Supplementary Table 3. Classification of clove repeat sequences.

Class	Superfamily	Lineage	Count	Length (bp)	Percentage of assembly length
Retrotransposons			6,287	80,731,053	21.80%
	LTR Copia		2,811	29,874,465	8.07%
		Ale	609	6,008,482	1.62%
		Alesia	40	322,247	0.09%
		Angela	97	996,594	0.27%
		Bianca	30	251,488	0.07%
		Gymco-IV	1	15,732	0.00%
		Ikeros	486	5,387,472	1.46%
		Ivana	323	2,875,238	0.78%
		SIRE	502	6,105,662	1.65%
		TAR	241	2,111,029	0.57%
		Tork	475	5,715,311	1.54%
		Unknown	7	85,210	0.02%
	LTR Gypsy		3,367	48,891,039	13.20%
		Athila	105	1,095,776	0.30%
		CRM	387	3,358,133	0.91%
		Galadriel	77	704,126	0.19%
		non-chromo-outgroup	5	168,216	0.05%
		Ogre	1,244	23,362,484	6.31%
		Reina	7	79,513	0.02%
		Tekay	1,538	20,015,880	5.41%
		Unknown	4	106,911	0.03%
	LTR Unknown		109	1,965,549	0.53%
DNA transposons	MITE		2,586	499,855	0.14%
	Helitron		1,059	30,022	0.01%
Tandem repeat			134,280	3,174,970	0.86%
Repeat element (unclassified)			123,383	76,254,065	20.59%
Total			279,940	160,689,965	43.40%

Supplementary Table 4. Classification of eucalyptus Copia and Gypsy LTR-retrotransposons.

Order	Superfamily	Lineage	Count	Length (bp)	Percentage of assembly length (690Mb)
LTR	Copia		7,736	88,014,391	12.73%
		Ale	1,302	13,907,418	2.01%
		Alesia	72	7,32,803	0.11%
		Angela	118	1,177,594	0.17%
		Bianca	102	982,202	0.14%
		Gymco-I	1	25,313	0.00%
		Ikeros	1,660	18,297,078	2.65%
		Ivana	428	4,851,223	0.70%
		SIRE	2,646	31,180,014	4.51%
		TAR	369	3,778,579	0.55%
		Tork	1,004	12,476,408	1.80%
		Unknown	34	605,759	0.09%
	Gypsy		2,910	38,170,125	5.52%
		Athila	104	1,526,850	0.22%
		Chromo-unclass	1	21,025	0.00%
		CRM	249	2,688,555	0.39%
		Galadriel	303	3,386,074	0.49%
		Non-chromo-outgroup	8	169,043	0.02%
		Ogre	719	11,205,165	1.62%
		Reina	2	19,239	0.00%
		Tekay	1,511	18,952,691	2.74%
		Unknown	13	201,483	0.03%
	Unknown		16	397,895	0.06%
	Total		10,662	126,582,411	18.31%

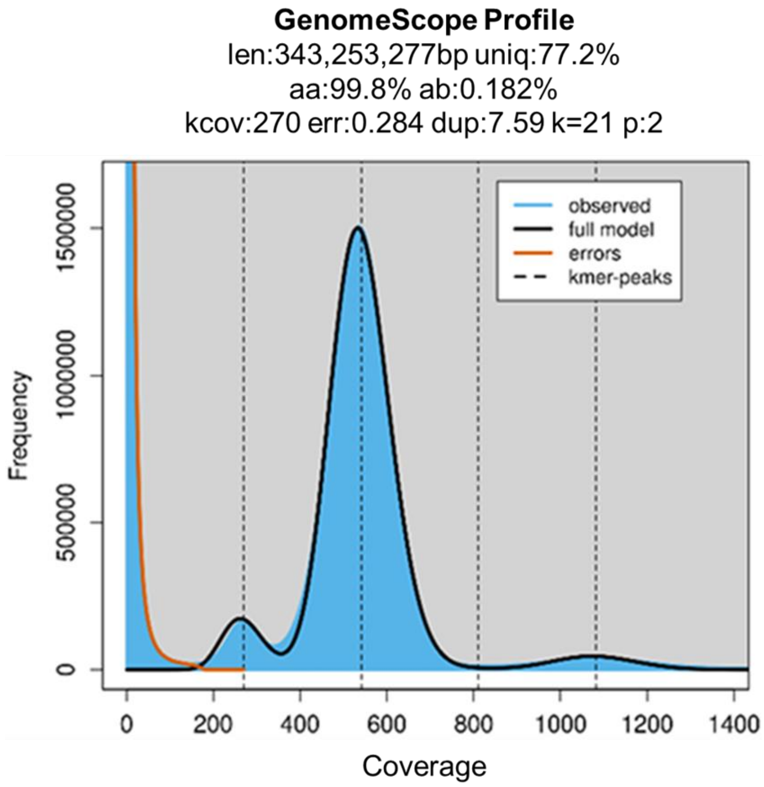

Supplementary Figure 1. GenomeScope K-mer spectra and fitted models for *Syzygium aromaticum*.

Cleaned Illumina PE reads from DNAseq libraries were analyzed by GenomeScope 2.0 to estimate genome size and percentage heterozygosity[1]. The software was run with a k-mer size equal to 21 and a ploidie level equal to 2. The resulting k-mer analysis indicated that the clove genome has a heterozygotie rate of 0.18%, and an estimated genome size of 343 Mbp.

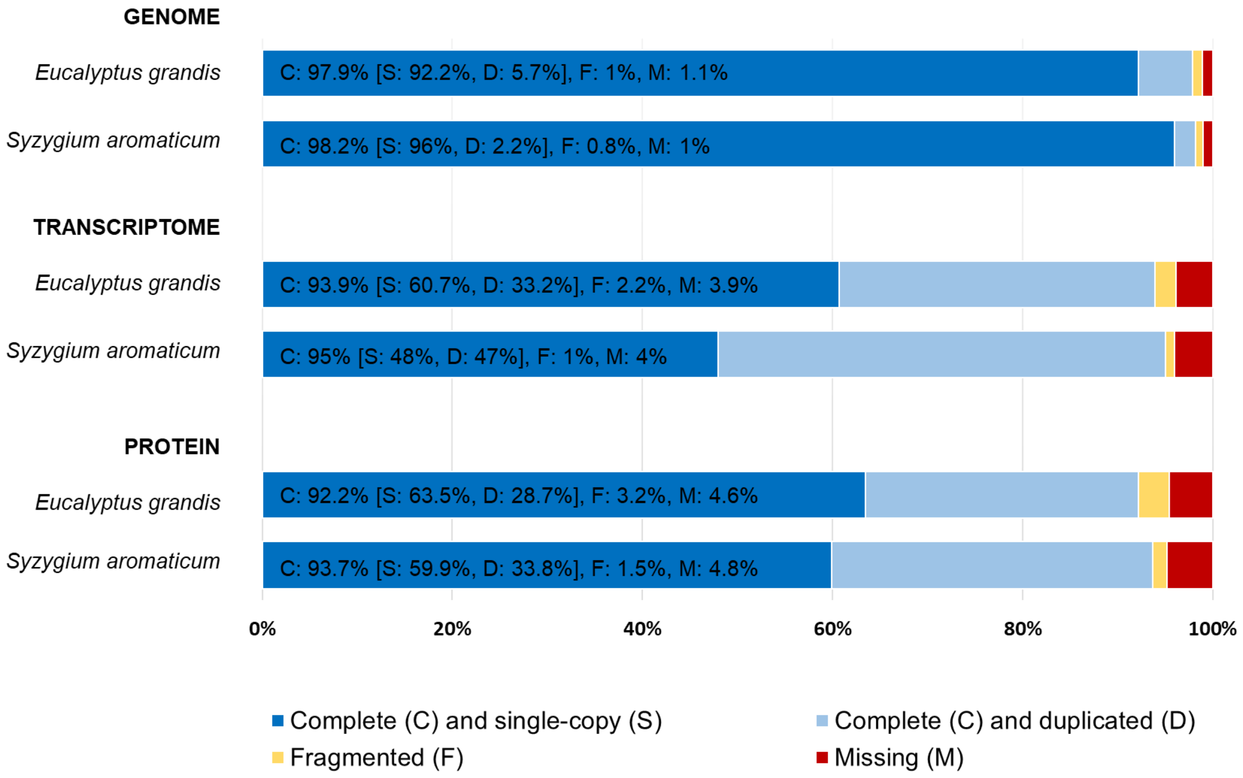

Supplementary Figure 2. BUSCO assessment of the *S. aromaticum* genome.

BUSCO results generated in the genome, transcriptome, and protein mode by using *Eucalyptus grandis* genome v.2 and the S*. aromaticum* final assembly (chromosome-level), predicted transcript and protein sets (BUSCO version 5.2.2 - dataset: eudicots_odb10, n=2326)[2].

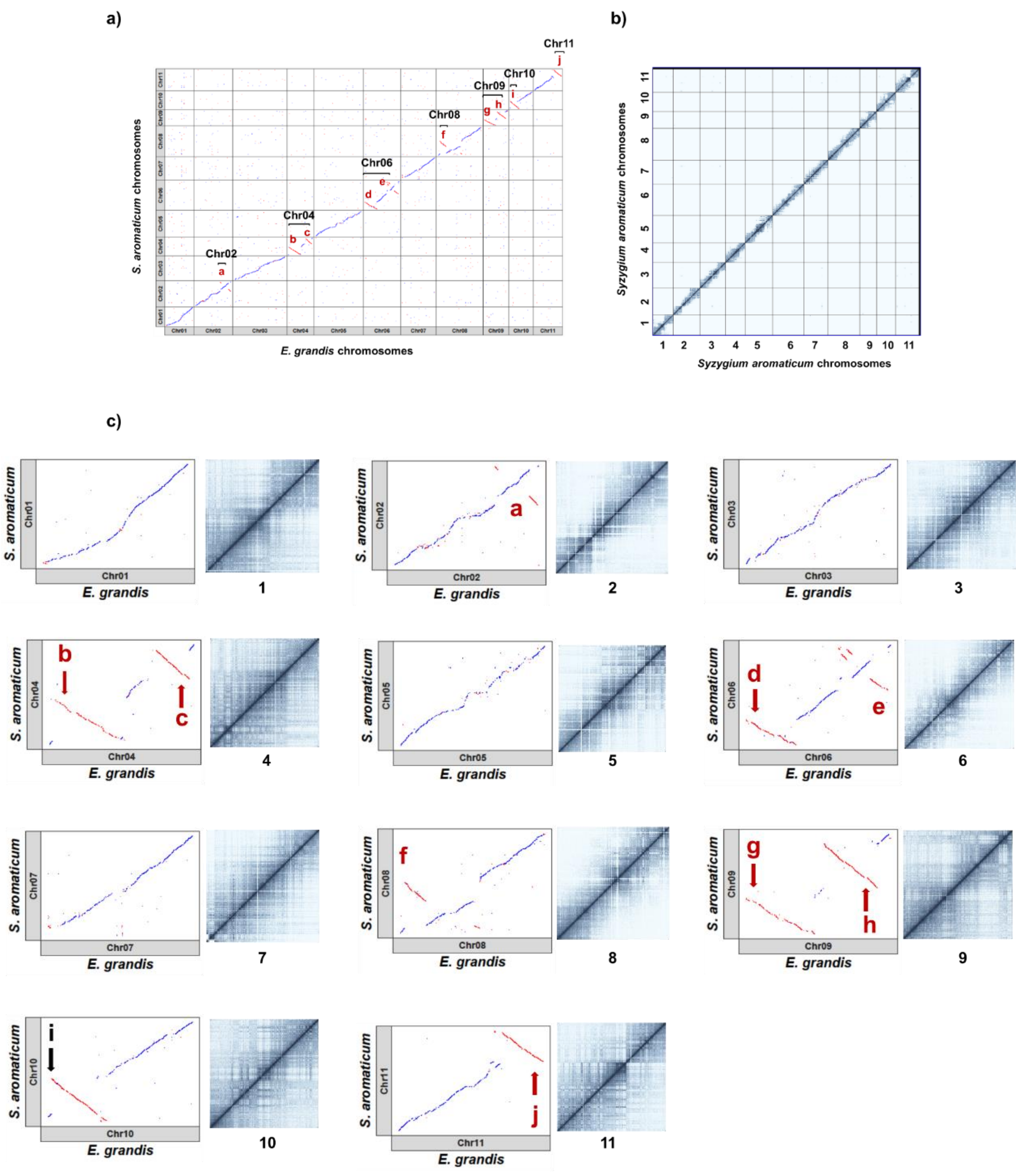

Supplementary Figure 3. Visualization of the Hi-C data used for assembling *Syzygium aromaticum* genome into 11 chromosomes.

a) DNA alignment of *Syzygium aromaticum* and *Eucalyptus grandis* 11 chromosomes. Letters (a to j) indicate the presence of intrachromosomal rearrangements in the chromosomes 2, 4, 6, 8, 9, 10. and 11 of *S. aromaticum* when compared to the corresponding chromosome of *E. grandis*. **b)** Hi-C contact maps showing the Hi-C interactions among the 11 assembled chromosomes of *S. aromaticum.* Dark signal indicates a higher contact probability. **c)** Detailed Hi-C contact maps for individual *S. aromaticum* chromosomes and the DNA alignment plot for the corresponding chromosome of *E. grandis.* On the Hi-C contact maps, the diagonal

indicates a strong Hi-C intrachromosomal signal among the *S. aromaticum* chromosomes and a very low interchromosomal Hi-C signal. This strong signal implies that there is high number of Hi-C data supporting the proposed scaffolding of contigs into the 11 *S. aromaticum* chromosomes.

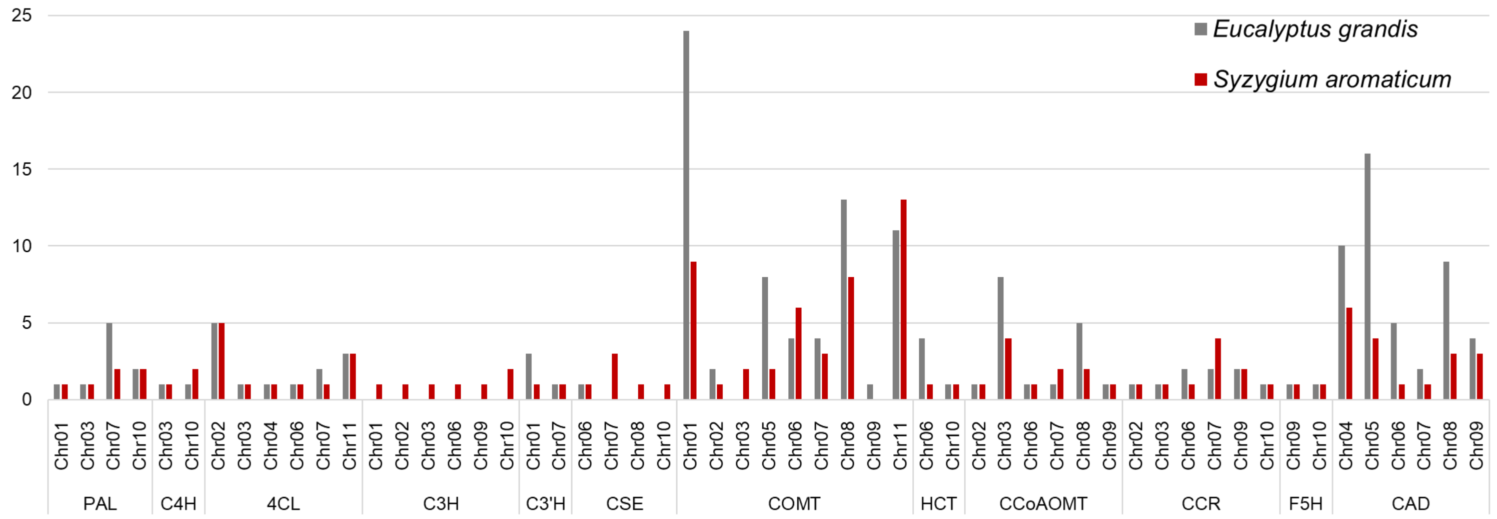

GENE FAMILY	PAL	C4H	4CL	C3H	C3'H	CSE	COMT	HCT	CCoAOMT	CCR	F5H	CAD
E. grandis	9	2	13	-	4	1	67	5	17	9	2	46
S. aromaticum	6	3	12	7	2	6	44	2	11	10	2	18

Supplementary Figure 4. Distribution and number of phenylpropanoid genes in the *S. aromaticum* genome**.**

Distribution across the 11 chromosomes (Chr) and number of phenylpropanoid genes found in *S. aromaticum*, and comparison with those reported for *E. grandis* [3,4].

PAL, phenylalanine ammonia-lyase; **C4H**, cinnamate 4-hydroxylase; **C3H**, 4-coumarate 3-hydroxylase; **COMT**, caffeate/5-hydroxyferulate 3-*O*-methyltransferase; **F5H**, ferulate 5-hydroxylase/coniferaldehyde 5-hydroxylase; **4CL**, 4-hydroxycinnamate:CoA ligase; **HCT**, 4-hydroxycinnamoyl CoA:shikimate/quinate hydroxycinnamoyltransferase; **C3′H**, 4-coumaroyl shikimate/quinate 3′-hydroxylase; **CSE**, caffeoyl shikimate esterase; **CCoAOMT**, caffeoyl CoA 3-*O*-methyltransferase; **CCR**, cinnamoyl CoA reductase; **CAD**, cinnamyl alcohol dehydrogenase

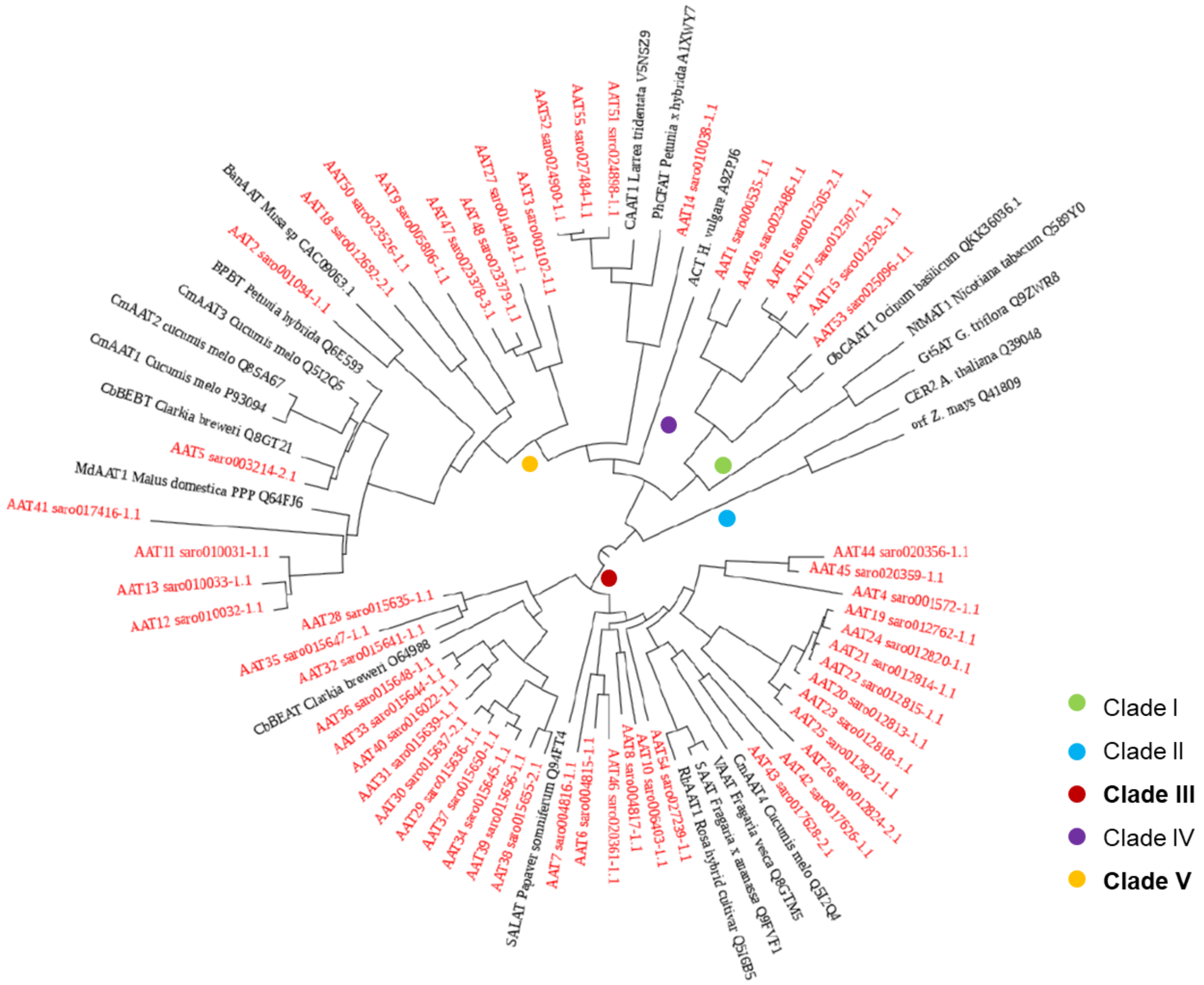

Supplementary Figure 5. Evolution of selected *S. aromaticum* putative alcohol acyl transferases (AAT).

Phylogenetic tree of *S. aromaticum* putative AAT protein sequences. The phylogenetic trees were generated by using Clustal Omega. Uniprot entry identifiers are indicated for the reference proteins (NCBI identifiers are indicated for CAC09063.1 and QKK36036.1). The alcohol acyltransferase from *Petunia hybrida* (PhCFAT), Larrea tridentata (CAAT1) and *Ocimum basilicum* (ObCAAT1), have been shown to catalyze the formation of coniferyl acetate from coniferyl alcohol and acetyl CoA. Clade I to V from D'Auria 2006[5].

Supplementary Note. Identification of gene from the BAHD superfamily and PIP families involved in the biosynthesis of eugenol.

The two final steps of the eugenol biosynthesis are catalyzed by biosynthetic genes belonging to families containing some members involved in the production of volatile phenylpropenes (BAHD superfamily and PIP family). Coniferyl alcohol is converted to coniferyl acetate by an alcohol acyltransferase (AAT) belonging to the BAHD (benzyl alcohol-acetyl-, anthocyanin-*O*-hydroxy-cinnamoyl-, anthranilate-N-hydroxycinnamoyl/benzoyl-, or deacetyl-vindoline) acyltransferase superfamily. Then, coniferyl acetate is used as a substrate for eugenol synthesis by eugenol synthase (EGS), an NADPH-dependent reductase belonging to the pinoresinol–lariciresinol reductase, isoflavone reductase, phenylcoumaran benzylic ether reductase (PIP) family[5,6]. Previous studies on clove EO produced from buds and leaves at different growth phases showed that eugenol acetate is the major constituent of the EO produced in young leaves and buds and that the eugenol content of EO increases during the development of the two organs, while the percentage of eugenol acetate decreases[7,8]. In previous studies, recombinant AAT from apple (MdAAT1), wild strawberry (VAAT), and cultivated strawberry (SAAT) exhibited catalytic activity with eugenol when tested in vitro as a substrate, and MdAAT1 also exhibited catalytic activity with isoeugenol and isochavicol as substrates[9,10]. We, therefore, evaluated AATs as good candidates not only for biosynthesis of eugenol from coniferyl acetate but also for biosynthesis of eugenol acetate from eugenol.

BADH proteins are promiscuous, making functional prediction from predicted amino acid sequences very difficult[5]. To identify the AAT genes involved in the biosynthesis of eugenol and eugenol acetate, we used reference proteins, including the characterized coniferyl alcohol acyltransferase from *Petunia hybrida* (CFAT) and *Ocimum basilicum* (ObCAAT1), and the cinnamyl alcohol acyltransferase from *Larrea tridentata* (LtCAAT1), which have been shown to catalyze the formation of coniferyl acetate from coniferyl alcohol and acetyl CoA[11-13]. We also used members of the BAHD superfamily belonging to classes III and V known to be involved in the biosynthesis of volatile esters[5].

EGS is a key enzyme for biosynthesis of eugenol and has been characterized in a few species. To identify putative EGS in clove genome we used the protein sequences of characterised EGS from eight different plant families in a phylogenetic analysis (Supplementary data set 2). For instance, EGS isolated from basil (ObEGS1) and *Clarkia breweri* (CbEGS1 and CbEGS2) catalyzes eugenol production by using coniferyl acetate as a substrate. The characterized *C. breweri* IGS1 and EGS1 have a very high percentage similarity (96%) but catalyze the production of isoeugenol and eugenol, respectively[14]. The EGS isolated from strawberry (FaEGS2) can catalyze the production of both

eugenol and isoeugenol (with a lower catalytic efficiency) from coniferyl acetate. Other members of the PIP family, such as the allyl-phenylpropene synthase (APS) isolated from creosote bush (*L. tridentata*), catalyze the conversion of *p*-coumaryl acetate and coniferyl acetate into chavicol and eugenol, respectively[6].

REFERENCES

1 Ranallo-Benavidez, T. R., Jaron, K. S. & Schatz, M. C. GenomeScope 2.0 and Smudgeplot for reference-free profiling of polyploid genomes. *Nature Communications* **11**, 1-10 (2020).

2 Simão, F. A., Waterhouse, R. M., Ioannidis, P., Kriventseva, E. V. & Zdobnov, E. M. BUSCO: assessing genome assembly and annotation completeness with single-copy orthologs. *Bioinformatics* **31**, 3210-3212 (2015).

3 Myburg, A. A. *et al.* The genome of *Eucalyptus grandis*. *Nature* **510**, 356-362, doi:10.1038/nature13308 (2014).

4 Carocha, V. *et al.* Genome-wide analysis of the lignin toolbox of *Eucalyptus grandis*. *New Phytol.* **206**, 1297–1313 (2015).

5 D'Auria, J. C. Acyltransferases in plants: a good time to be BAHD. *Curr. Opin. Plant Biol.* **9**, 331–340 (2006).

6 Koeduka, T. Functional evolution of biosynthetic enzymes that produce plant volatiles. *Biosci. Biotechnol. Biochem.* **82**, 192–199 (2018).

7 Razafimamonjison, G. *et al.* Variations in yield and composition of leaf essential oil from *Syzygium aromaticum* at various phases of development. *Int. J. Basic Appl. Sci.* **5**, 90 (2016).

8 Razafimamonjison, G., Jahiel, M., Ramanoelina, P., Fawbush, F. & Danthu, P. Effects of phenological stages on yield and composition of essential oil of *Syzygium aromaticum* buds from Madagascar. *Int. J. Basic Appl. Sci.* **2**, 312–318 (2013).

9 Yauk, Y. K. *et al.* Alcohol acyl transferase 1 links two distinct volatile pathways that produce esters and phenylpropenes in apple fruit. *Plant J.* **91**, 292–305 (2017).

10 Beekwilder, J. *et al.* Functional characterization of enzymes forming volatile esters from strawberry and banana. *Am. Soc. Plant Biol.* **135**, 1865–1878 (2004).

11 Dhar, N. *et al.* Characterization of a sweet basil acyltransferase involved in eugenol biosynthesis. *J. Exp. Bot.* **71**, 3638–3652 (2020).

12 Kim, S. J. *et al.* Allyl/propenyl phenol synthases from the creosote bush and engineering production of specialty/commodity chemicals, eugenol/isoeugenol, in *Escherichia coli*. *Arch. Biochem. Biophys.* **541**, 37–46 (2014).

13 Dexter, R. *et al.* Characterization of a petunia acetyltransferase involved in the biosynthesis of the floral volatile isoeugenol. *Plant J.* **49**, 265–275 (2007).

14 Koeduka, T. *et al.* The multiple phenylpropene synthases in *both Clarkia* breweri and *Petunia hybrida* represent two distinct protein lineages. *Plant J.* **54**, 362–374, doi:10.1111/j.1365-313X.2008.03412.x (2008).
