# Supplementary material for: The clove (Syzygium aromaticum) genome provides insights into the eugenol biosynthesis pathway
Source: Commun Biol. 2022 Jul 9;5:684. doi: 10.1038/s42003-022-03618-z (PMC9271057; doi:10.1038/s42003-022-03618-z)
Supplement: Supplementary file 2 — Description of Additional Supplementary Files [file 42003_2022_3618_MOESM2_ESM.pdf]

Description of Additional Supplementary Files

File name: Supplementary Data 1
Description: Illumina and Oxford Nanopore Technologies sequencing summaries.Number of Oxford Nanopore Technologies reads and Illumina sequencing reads (generated from DNAseq libraries, Hi-C libraries and RNASeq libraries) used to generate the S. aromaticum genome assembly.

File name: Supplementary Data 2
Description: List of proteins used to identify selected S. aromaticum gene families. List of the reference proteins used to identify member of 14 gene families (PAL, C4H, 4CL, HCT, C3'H, C3H, CSE, COMT, CCoAOMT, F5H, CCR, CAD, BADH and PIP) in S. aromaticum genome using a homology-based approach.

File name: Supplementary Data 3
Description: List of selected genes in S. aromaticum. List of selected S. aromaticum genes belonging to the 14 families PAL, C4H, 4CL, HCT, C3'H, C3H, CSE, COMT, CCoAOMT, F5H, CCR, CAD, BADH and PIP, and selected gene encoding for putative ABC transporters, esterase and hydrolase.

File name: Supplementary Data 4
Description: Additional HPLC-UV and GC-MS analysis results. Concentration values behind the figure 5 and concentrations of other compounds (not indicated on Figure 5) detected in clove organs analysed.
