# Supplementary material for: The clove (Syzygium aromaticum) genome provides insights into the eugenol biosynthesis pathway
Source: Commun Biol. 2022 Jul 9;5:684. doi: 10.1038/s42003-022-03618-z (PMC9271057; doi:10.1038/s42003-022-03618-z)
Supplement: Supplementary file 7 — Reporting Summary [file 42003_2022_3618_MOESM7_ESM.pdf]

Corresponding author(s): Nikolai V. Ivanov
Last updated by author(s): May 25, 2022

Reporting Summary

Nature Research wishes to improve the reproducibility of the work that we publish. This form provides structure for consistency and transparency in reporting. For further information on Nature Research policies, see our Editorial Policies and the Editorial Policy Checklist.

Statistics

For all statistical analyses, confirm that the following items are present in the figure legend, table legend, main text, or Methods section.

n/a	Confirmed	
☐	☒	The exact sample size (n) for each experimental group/condition, given as a discrete number and unit of measurement
☐	☒	A statement on whether measurements were taken from distinct samples or whether the same sample was measured repeatedly
☐	☒	The statistical test(s) used AND whether they are one- or two-sided<br>*Only common tests should be described solely by name; describe more complex techniques in the Methods section.*
☒	☐	A description of all covariates tested
☒	☐	A description of any assumptions or corrections, such as tests of normality and adjustment for multiple comparisons
☐	☒	A full description of the statistical parameters including central tendency (e.g. means) or other basic estimates (e.g. regression coefficient) AND variation (e.g. standard deviation) or associated estimates of uncertainty (e.g. confidence intervals)
☒	☐	For null hypothesis testing, the test statistic (e.g. F, t, r) with confidence intervals, effect sizes, degrees of freedom and P value noted<br>*Give P values as exact values whenever suitable.*
☒	☐	For Bayesian analysis, information on the choice of priors and Markov chain Monte Carlo settings
☒	☐	For hierarchical and complex designs, identification of the appropriate level for tests and full reporting of outcomes
☒	☐	Estimates of effect sizes (e.g. Cohen's d, Pearson's r), indicating how they were calculated

Our web collection on statistics for biologists contains articles on many of the points above.

Software and code

Policy information about availability of computer code

Data collection	MinKnow<br>Illumina Control Software
Data analysis	Basecalling: guppy 4.0.15<br>Genome profiling: GenomeScope 2.0<br>De novo genome assembly: seqkit 0.14.0, fastp 0.20.1, ratatosk 0.3.0, minimap2 2.17, miniasm 0.3_r179, minipolish 0.1.2, ntedit 1.3.2, purge_dups 1.2.5, bwa 0.7.17, samtools 1.11, yahs 1.0<br>Evaluation of the genome assembly: BUSCO 5.2.2<br>Genome annotation: fastp 0.23.2, minimap2 2.22, bedtools 2.30.0, samtools 1.14, scallop 0.10.5, taco 0.7.3, augustus 3.4.0, gffread 0.12.7, transdecoder 5.5.0, diamond 2.0.14, TEnester (fa95fb13950efef32a1746dd610dfc2cd2193955), TEsorter 1.3.0, GRF 1.0, EAHelitron 1.53, tantan 26, Red 2.0<br>Synteny analysis: MCScanX (97e74f40224368ffde3401b61f649d6acd897a27), SynVisio (80412dc9becb39cafd617f80017da63486125a54)<br>Phylogentic analysis: diamond 2.0.14, clustalo 1.2.4, fasttree 2.1.10, newick_utils 1.6, ete3 3.1.1<br>Differential gene expression analysis: star 2.7.10a, R 4.1.2, deseq2 1.34.0, pheatmap 1.0.12

For manuscripts utilizing custom algorithms or software that are central to the research but not yet described in published literature, software must be made available to editors and reviewers. We strongly encourage code deposition in a community repository (e.g. GitHub). See the Nature Research guidelines for submitting code & software for further information.

Data

Policy information about availability of data

All manuscripts must include a data availability statement. This statement should provide the following information, where applicable:

- Accession codes, unique identifiers, or web links for publicly available datasets
- A list of figures that have associated raw data
- A description of any restrictions on data availability

Illumina and Oxford Nanopore reads are available from the National Center for Biotechnology Information Short Read Archive (SRA) under accession: PRJNA660399. This Whole Genome Shotgun project has been deposited at DDBJ/ENA/GenBank under the accession JACTMA000000000. The version described in this paper is version JACTMA0100000000. The genome annotation is available from Zenodo under DOI: 10.5281/zenodo.6579856.

Field-specific reporting

Please select the one below that is the best fit for your research. If you are not sure, read the appropriate sections before making your selection.

☒ Life sciences ☐ Behavioural & social sciences ☐ Ecological, evolutionary & environmental sciences

For a reference copy of the document with all sections, see nature.com/documents/nr-reporting-summary-flat.pdf

Life sciences study design

All studies must disclose on these points even when the disclosure is negative.

Sample size	The clove genome was generated from a single tree considered as representative of the species.
Data exclusions	No data were excluded from the analysis
Replication	For the combined metabolome and transcriptome analysis, clove leaves and buds were sampled in triplicate for each growth phase
Randomization	Randomization was not relevant for the genomic, gene expression and metabolomic analyses performed in the studied.
Blinding	Blinding was not relevant for the comparison performed between clove leaves and buds at different stage of developments

Reporting for specific materials, systems and methods

We require information from authors about some types of materials, experimental systems and methods used in many studies. Here, indicate whether each material, system or method listed is relevant to your study. If you are not sure if a list item applies to your research, read the appropriate section before selecting a response.

Materials & experimental systems

n/a	Involved in the study	
☒	☐	Antibodies
☒	☐	Eukaryotic cell lines
☒	☐	Palaeontology and archaeology
☒	☐	Animals and other organisms
☒	☐	Human research participants
☒	☐	Clinical data
☒	☐	Dual use research of concern

Methods

n/a	Involved in the study	
☒	☐	ChIP-seq
☒	☐	Flow cytometry
☒	☐	MRI-based neuroimaging
